# Fungal symbionts of marine organisms: The case of *Cladosporium* and its bioactive secondary metabolites

**DOI:** 10.3934/microbiol.2026014

**Published:** 2026-06-08

**Authors:** Maria Michela Salvatore, Anna Andolfi, Rosario Nicoletti

**Affiliations:** 1 Department of Veterinary Medicine and Animal Production, University of Naples ‘Federico II’, 80137 Naples, Italy; 2 Department of Chemical Sciences, University of Naples ‘Federico II’, 80126 Naples, Italy; 3 Council for Agricultural Research and Economics, Research Centre for Olive, Fruit and Citrus Crops, 81100 Caserta, Italy

**Keywords:** marine fungi, Cladosporiaceae, chemodiversity, bioactive products, mycobiome

## Abstract

Fungi in the genus *Cladosporium* (Dothideomycetes, Cladosporiaceae) are widespread in every kind of natural and anthropic terrestrial habitats, where they are reported to be involved in ecological interactions. The developments in marine mycology have demonstrated that, rather than being typically terrestrial, they are also common in the sea. In this manuscript, we examined the synthetic potential of *Cladosporium* strains recovered from marine organisms and the available information concerning bioactivities of their secondary metabolites. As many as 106 compounds have been identified as products of these fungi so far, belonging to several classes of natural compounds, such as benzopyrones, diketopiperazines, flavonoids, lactones, macrolides, naphthalenones, pyrones, steroids, tetramic acids, and xanthones; 29 of them were first identified from this biological material, representing a valuable source of chemodiversity, which deserves to be investigated in-depth for the related biological properties in view of possible biotechnological exploitation.

## 1. Introduction

Fungi in the genus *Cladosporium* have a pervasive occurrence in all natural and anthropic environments on Earth, having been reported even from extreme habitats such as deep ocean vents [1], hypersaline lakes [2], Antarctic ice [3], and oil-polluted sea water [4]. In this extraordinary ecological adaptability, they have been found to establish symbiotic relationships with a varied set of organisms, ranging from plant pathogenicity to trophic support of mycophagous insects [5],[6]. These interactions are often mediated by bioactive secondary metabolites, which represent a trove of chemodiversity available for more accurate assessments in view of possible exploitation in biotechnologies. A review of the chemosynthetic aptitudes of *Cladosporium* strains recovered in association with marine organisms is presented in this manuscript to provide a comprehensive insight into the state of the art and applicative perspectives. The literature on *Cladosporium* spp. associated with marine organisms was searched based on a list of keywords, including ‘*Cladosporium*’, ‘marine organisms’, ‘sponges’, ‘algae’, and ‘secondary metabolites’, across PubMed, Google Scholar, Web of Science, SciFinder, and Scopus, covering research up to 28 February 2026.

## 2. Notes on *Cladosporium* taxonomy

The widespread occurrence of ascomycetes belonging to the genus *Cladosporium* (Dothideomycetes, Cladosporiales, Cladosporiaceae) reflects a high genetic variation, which is attested by over two hundred species described so far [7]. This number has been increasing following the exploration of biodiversity in diverse environments and peculiar ecological contexts [8]–[11]. Moreover, the use of molecular markers has enabled taxonomists to disentangle the intricate web within the three major species complexes (s.c.), namely *C. cladosporioides* s.c., *C. herbarum* s.c., and *C. sphaerospermum* s.c., in which this genus is traditionally sorted [12]. In this respect, researchers in the field should avoid limiting their identifications to an anonymous ‘*Cladosporium* sp.’, as was found in most of the research considered in this review, or to rely on the use of rDNA-ITS sequences only; rather, it is recommended to try to scrupulously follow the official criteria for a correct classification based on sequencing more informative markers, such as the translation elongation factor 1-alpha and the actin genes [12].

The application of these basic taxonomic methods has brought the description of new *Cladosporium* species from marine sources. Particularly, isolations carried out from samples collected along the coasts of South Korea have afforded the identification of *C. snafimbriatum* in the *C. herbarum* s.c., *C. marinisedimentum* in the *C. sphaerospermum* s.c., *C. lagenariiforme*, *C. maltirimosum*, and *C. marinum* in the *C. cladosporioides* s.c., with the latter three recovered from unspecified seaweeds [13]. Moreover, another new species in the *C. cladosporioides* s.c., *C. rubrum*, was identified from a green alga (*Ulva* sp.) collected in the estuary of Ria de Aveiro in Portugal [14].

## 3. Symbiotic occurrence of *Cladosporium* at sea

As for other fungi associated with marine organisms, little is known about the biological significance of symbioses involving *Cladosporium*. The increasing concern on the opportunistic pathogenic behavior of these fungi [12] has involved sea turtles, following the isolations of *C. cladosporioides*, *C. anthropophilum*, and other unidentified species from internal organs of individuals affected by phaeohyphomycosis [15]–[18]. Pathological effects have also been hypothesized on corals (*Porites lutea*) affected by the pink-line syndrome [19], as well as in the case of findings concerning the octopus *Eledone cirrhosa* [20] and holothurians [21]. Conversely, a strain of *C. halotolerans* was found to induce protective effects by mitigating tissue loss at elevated temperature when inoculated on the scleractinian coral *Stylophora pistillata* [22]. Indeed, their constant feature of being part of the mycobiome of corals [23],[24] supports the inference that, rather than being derived by the occasional transport of spores by the marine streams, these fungi could play a functional role in these and other sessile marine organisms. In this respect, *Cladosporium* was the most represented fungus in investigations based on specimens of sponges, namely three species collected along the coast of Western Ireland [25] and four species collected at the island of Ischia, Italy [26]. Moreover, out of 37 *Cladosporium* isolates from miscellaneous organisms collected along the Australian coasts, 20 were obtained from sponges of unidentified species [27].

Fungi in the genus *Cladosporium* have also been detected as a common component of mycoplankton in marine waters in investigations carried out in geographical contexts, such as the coastal ecosystems of the South China Sea [28],[29], the Yellow Sea [30], and the English Channel [31]. Even if not being symbiotic in the strict sense, this occurrence is not merely occasional; in fact, these fungi have been determined to perform a saprotrophic functional role in processing algal polysaccharides, making them involved in the trophic transfer of organic matter in the marine food webs [32].

This role is also played by *Cladosporium* strains associated with macroalgae, as demonstrated in the case of strains from three species collected from *Fucus* sp. at two locations in the Netherlands [33]. Starting with the pioneering finding of *C. algarum* (currently known as *C. macrocarpum*) on decaying *Laminaria digitata* [34], their widespread occurrence has resulted in several investigations concerning these marine plants, as also attested by the above-mentioned identification of novel species. Particularly, in the investigation carried out in an estuarine environment in Portugal leading to the identification of *C. rubrum*, fungi in the genus *Cladosporium* were the most abundant ascomycetes with 147 out of 486 isolates (corresponding to 30.2%) [14]. Again, *Cladosporium* was the most frequent fungus recovered from algae of undetermined species during an extensive survey on marine fungi in coastal and estuarine Portuguese environments, with 85 of 129 isolates [35]. Moreover, *Cladosporium* was the most abundant fungal genus with 24 isolates recovered from four species of macroalgae in Antarctica; of 25 isolates obtained from the brown seaweed *Adenocystis utricularis*, as many as 20 were *Cladosporium* [36]. In addition, it was the second most abundant genus of fungi recovered from the red alga *Palmaria palmata* at a location in the White Sea, Russia [37], and the third one from other brown algae, namely *Fucus serratus*, by the German island of Helgoland [38] and *Sargassum thunbergii* in South Korea [39]. Finally, one of the most common in isolations was carried out from red algae of the genus *Kappaphycus* in the Philippines [40].

In addition to the above-mentioned large-scale investigations, the widespread ecological association as symbionts of marine organisms is well depicted by the long list of occasional findings of single or groups of isolates reported in Tables 1 and 2.

**Table 1.** Occurrence of Cladosporium in association with marine plants.

Organism	Species	Location
Angiospermae
*Cymodocea serrulata*	*Cladosporium* sp.	Tamil Nadu, India [41]
*Cymodocea* sp.	*Cladosporium* sp.	
*Enhalus acoroides*	*Cladosporium* sp., *C. sphaerospermum*	Tarai island, Thailand [42]
*Halodule* sp.	*Cladosporium* sp.	Tamil Nadu, India [41]
*Halodule wrightii*	*Cladosporium* sp.	East Cove lagoon, Florida [43]
*Halophila ovalis*	*Cladosporium* sp.	Tamil Nadu, India [44]
	*C. cladosporioides*, *C. sphaerospermum*	Pakmeng, Thailand [45]
*Phyllospadix scouleri*	*Cladosporium* sp.	San Juan island, Washington [46]
*Posidonia oceanica*	*C. cladosporioides*, *C. cucumerinum*, *C. herbarum*, *C. oxysporum*, *C. sphaerospermum*, *Cladosporium* sp.	Riva Trigoso bay, Italy [47]
	*C. aggregatocicatricatum*, *C. allicinum*, *C. cladosporioides*, *C. herbarum*, *C. pseudocladosporioides*, *C. velox*, *C. xylophilum*	Elba island, Italy [48]
	*C. tenellum*	Giglio isle, Italy [49]
	*Cladosporium* sp.	Northern Sicily, Italy [50]
	*C. aggregatocicatricatum*, *Cladosporium* sp.	Konnos bay, Cyprus [51]
*Syringodium* sp.	*Cladosporium* sp.	Tamil Nadu, India [41]
*Thalassia hemprichii*	*Cladosporium* sp.	Pakmeng, Thailand [45]
*Thalassia* sp.	*Cladosporium* sp.	Tamil Nadu, India [41],[44]
*Thalassia testudinum*	*Cladosporium* sp.	East Cove lagoon, Florida [43]
	*Cladosporium* sp.	Hobie island, Florida [52]
*Zostera japonica*	*Cladosporium* sp.	San Juan island, Washington [46]
*Zostera marina*	*Cladosporium* sp.	Chesapeake bay, Maryland [53]
	*Cladosporium* sp.	San Juan island, Washington [46]
	*Cladosporium* sp.	Bodega bay, California [54]
	*C. halotolerans*, *Cladosporium* sp.	Kiel fjord, Germany [55]
Chlorophyta
*Acrosiphonia arcta*	*Cladosporium* sp., *C. tenuissimum*	Elephant island, Antacrtica [56]
*Caulerpa racemosa*	*Cladosporium* sp.	Tamil Nadu, India [44],[57]
*Caulerpa sertularioides*	*Cladosporium* sp.	various locations [58] Tamil Nadu, India [57]
	*Cladosporium* sp.	Vera Cruz, Mexico [59]
*Chaetomorpha antennina*	*Cladosporium* sp.	Tamil Nadu, India [60]
*Flabellia petiolata*	*C. allicinum*, *C. cladosporioides*, *C. herbarum*, *C. sphaerospermum*	Elba island, Italy [61]
*Halimeda macroloba*	*Cladosporium* sp.	Tamil Nadu, India [60]
*Monostroma hariotii*	*Cladosporium* sp.	Antarctica [62]
*Pseudendoclonium submarinum*	*C. cladosporioides*	Ipswich, Massachusetts [63]
*Rhipocephalus phoenix*	*Cladosporium* sp.	Vera Cruz, Mexico [59]
*Ulva lactuca*	*Cladosporium* sp.	Tamil Nadu, India [44],[57],[60]
*Ulva* sp.	*C. cladosporioides*	Abou-keer, Egypt [64]
	*C. perangustum*, *C. rubrum*	Ria Aveiro, Portugal [14]
unidentified species	*Cladosporium* sp.	Australia [27]
	*Cladosporium* sp.	Palk bay, India [65]
Rhodophyta
*Acanthophora spicifera*	*Cladosporium* sp.	Andaman islands, India [66]
*Actinotrichia fragilis*	*Cladosporium* sp.	Okinawa, Japan [67]
*Asparagopsis taxiformis*	*C. cladosporioides*, *C. pseudocladosporioides*	Linosa, Italy [68]
*Curdiea racovitzae*	*Cladosporium* sp.	Antarctica [62]
*Devaleraea ramentacea*	*Cladosporium* sp.	Bay of Fundy, Canada [58]
*Gelidiella acerosa*	*Cladosporium* sp.	Tamil Nadu, India [60]
*Georgiella confluens*	*Cladosporium* sp.	Antarctica [62]
*Gracilaria corticata*	*Cladosporium* sp.	Tamil Nadu, India [60]
*Gracilaria edulis*	*Cladosporium* sp.	
*Gracilaria salicornia*	*Cladosporium* sp.	
*Gracilaria* sp.	*C. halotolerans*	Bay of Bengal, Bangladesh [69]
*Gracilariopsis longissima*	*Cladosporium* sp.	Suez canal, Egypt [70]
*Grateloupia lithophila*	*Cladosporium* sp.	Tamil Nadu, India [44],[57]
*Halymenia* sp.	*Cladosporium* sp.	various locations [58]
	*Cladosporium* sp.	Tamil Nadu, India [57]
*Kappaphycus alvarezii*	*Cladosporium* sp.	Calatagan, Philippines [40]
*Kappaphycus striatus*	*Cladosporium* sp.	
*Laurencia okamurae*	*C. cladosporioides*	Qingdao, China [71]
*Neopyropia yezoensis*	*Cladosporium* sp.	Lianyungang, China [72]
*Neuroglossum delesseriae*	*Cladosporium* sp.	De Mayo island, Antarctica [36]
*Palmaria decipiens*	*Cladosporium* sp.	
*Palmaria palmata*	*C. ramotenellum*	Batz-sur-Mer, France [73]
	*C. cladosporioides*, *C. fusiforme*, *C. halotolerans*, *C. herbarum*, *C. salinae*, *C. sphaerospermum*	Kandalaksha, Russia [37]
*Plocamium cartilagineum*	*Cladosporium* sp.	Shetland islands, Scotland [58]
*Portieria hornemannii*	*Cladosporium* sp.	Tamil Nadu, India [44],[57]
*Pterocladia* sp.	*C. cladosporioides*, *C. macrocarpum*	Abou-keer, Egypt [64]
*Pyropia endiviifolia*	*Cladosporium* sp.	Antarctica [62]
*Sarcopeltis skottsbergii*	*C. halotolerans*	De Mayo island, Antarctica [36]
unidentified species	*Cladosporium* sp.	Australia [27]
Heterokonta–phaeophyceae
*Adenocystis utricularis*	*C. cladosporioides*, *C. sphaerospermum*, *Cladosporium* sp.	De Mayo island, Antarctica [36]
*Ascophyllum nodosum*	*Cladosporium* sp.	Shetland islands, Scotlans [58]
*Ascoseira mirabilis*	*Cladosporium* sp.	Antarctica [74]
*Colpomenia sinuosa*	*Cladosporium* sp.	Andaman islands, India [66]
*Dictyota dichotoma*	*C. uredinicola*	Mypadu beach, India [75]
	*C. allicinum*	Giglio island, Italy [49]
*Ericaria mediterranea*	*C. macrocarpum* (identified as *C. algarum*)	Gulf of Naples, Italy [76]
*Fucus serratus*	*Cladosporium* sp.	Helgoland, Germany [38]
*Fucus* sp.	*C. europaeum*, *C. ramotenellum*, *C. sphaerospermum*	Netherlands [33]
*Fucus spiralis*	*C. cladosporioides*	Ria Aveiro, Portugal [14]
*Laminaria digitata*	*C. macrocarpum* (identified as *C. algarum*)	unknown [34]
*Padina gymnospora*	*Cladosporium* sp.	various locations [58]
	*Cladosporium* sp.	Tamil Nadu, India [57]
*Padina pavonica*	*C. allicinum*, *C. bruhnei*, *C. cladosporioides*, *C. delicatulum*, *C. halotolerans*, *C. iranicum*, *C. pseudocladosporioides*, *C. ramotenellum*, *C. sphaerospermum*, *C. subtilissimum*, *C. uredinicola*, *C. xylophilum*, *Cladosporium* sp.	Elba island, Italy [25]
*Sargassum ilicifolium*	*Cladosporium* sp.	Andaman islands, India [66]
*Sargassum* sp.	*Cladosporium* sp.	Tamil Nadu, India [44]
*Sargassum thunbergii*	*C. anthropophilum*, *C. cladosporioides*, *C. halotolerans*, *C. ramotenellum*, *C. tenuissimum*	South Korea [39]
*Sargassum wightii*	*C. cladosporioides*	Tamil Nadu, India [77]
	*Cladosporium* sp.	Tamil Nadu, India [44],[57]
*Stoechospermum polypodioides*	*Cladosporium* sp.	Tamil Nadu, India [60]
*Turbinaria conoides*	*Cladosporium* sp.	various locations [58]
	*Cladosporium* sp.	Tamil Nadu, India [57]
*Turbinaria* sp.	*Cladosporium* sp.	various locations [58]
	*Cladosporium* sp.	Tamil Nadu, India [57]
unidentified species	*Cladosporium* sp.	Australia [27]
	*Cladosporium* sp.	King George island, Antarctica [78]
unspecified seaweeds	*C. halotolerans*, *C. lagenariiforme*, *C*. *maltirimosum*, *C. marinum*, *C. perangustum*, *C. proteacearum*, *C. rectoides*, *C. tenuissimum*	South Korea [13]

**Table 2.** Occurrence of *Cladosporium* in association with marine animals.

Organism	Species	Location
Porifera
*Agelas citrina*	*C. cladosporioides*, *C. macrocarpum*, *C. tenuissimum*	El Ein El-Soukhna, Egypt [79]
*Agelas oroides*	*C. aciculare*, *C. aggregatocicatricatum*, *C. angustisporum C. dominicanum*, *C. halotolerans*, *C. limoniforme*, *C. longicatenatum*, *C. perangustum*, *C. ramotenellum*, *C. sphaerospermum*, *Cladosporium* sp., *C. tenellum*	Israel [80]
*Amphilectus digitata*	*C. atroseptum*, *C. brevicompactum*, *C. cladosporioides*, *C. sphaerospermum*	Sakhalin island, Russia [81]
*Amphimedon viridis*	*Cladosporium* sp.	São Sebastiao, Brazil [82]
*Aplysina aerophoba*	*C. herbarum*	Banyuls sur Mer, France [83]
	*Cladosporium* sp.	Tenerife, Spain [84]
*Aplysina cavernicola*	*C. cladosporioides*, *C. delicatulum*, *C. perangustum*, *C. pseudocladosporioides*	Villefranche sur mer, France [85]
*Callyspongia aerizusa*	*C. herbarum*	Bali, Indonesia [86]
*Callyspongia* sp.	*C. halotolerans*	Eilat, Israel [87]
	*Cladosporium* sp.	Xieyang island, China [88]
*Callyspongia vaginalis*	*Cladosporium* sp.	Dominica [84]
*Celtodoryx girardae*	*C. cladosporioides*	El Ein El-Soukhna, Egypt [79]
*Chondrilla nucula*	*Cladosporium* sp.	Ischia island, Italy [26]
*Chondrilla* sp.	*C. halotolerans*	Andaman Sea, India [87]
*Chondrosia reniformis*	*Cladosporium* sp.	Ischia island, Italy [26]
*Cliona celata*	*C. cladosporioides*, *C. sphaerospermum*	El Ein El-Soukhna, Egypt [79]
*Cliona* sp.	*C. cladosporioides*	Los Molles, Chile [89]
*Cliona viridis*	*Cladosporium* sp.	Rameswaram, India [90]
*Crambe crambe*	*C. pseudocladosporioides*	Villefranche sur mer, France [85]
	*Cladosporium* sp.	Ischia island, Italy [26]
*Dactylospongia* sp.	*C. halotolerans*	Mandeh island, Indonesia [91]
*Dragmacidon reticulatum*	*Cladosporium* sp.	São Sebastiao, Brazil [82]
*Dysidea fragilis*	*C. aggregatocicatricatum*, *C. allicinum, C. cladosporioides*, *C. perangustum*, *C. pseudocladosporioides*, *C. psychrotolerans*, *C. subtilissimum*, *C. xylophilum*	Gurraig Sound, Ireland [25]
*Ectyplasia perox*	*Cladosporium* sp.	Dominica [84]
*Grantia compressa*	*C. allicinum*, *C. cladosporioides*, *C. pseudocladosporioides*	Coranroo, Ireland [92]
*Halichondria panicea*	*C. atroseptum*	Sakhalin island, Russia [81]
*Haliclona melana*	*C. cladosporioides*	São Sebastiao, Brazil [93]
*Hyrtios* sp.	*Cladosporium* sp.	Red Sea [94]
*Ircinia oros*	*Cladosporium* sp.	Malta [84]
*Mandracis mirabilis*	*C. cladosporioides*, *C. oxysporum*	El Ein El-Soukhna, Egypt [79]
*Mycale laxissima*	*Cladosporium* sp.	São Sebastiao, Brazil [82]
*Myxilla incrustans*	*Cladosporium* sp.	Helgoland, Germany [84]
*Niphates rowi*	*Cladosporium* sp.	Gulf of Aqaba, Israel [95]
*Oscarella lobularis*	*C. tenuissimum*	El Ein El-Soukhna, Egypt [79]
	*Cladosporium* sp.	Tenerife, Spain [84]
*Pachymatisma johnstonia*	*C. allicinum*, *C. cladosporioides*, *C. halotolerans*, *C. pseudocladosporioides*, *C. subuliforme*	Gurraig Sound, Ireland [25]
*Petrosia ficiformis*	*Cladosporium* sp.	Ischia island, Italy [26]
*Phorbas tenacior*	*C. cladosporioides*, *C. perangustum*, *C. pseudocladosporioides*, *C. ramotenellum*	Villefranche sur mer, France [85]
*Plakina* sp.	*Cladosporium* sp.	Andaman Sea, India [87]
*Spongosorites difficilis*	*C. tenuissimum*	El Ein El-Soukhna, Egypt [79]
*Stelligera rigida*	*C. herbarum*	
*Sycon ciliatum*	*C. allicinum*, *C. cladosporioides*, *C. halotolerans*	Coranroo, Ireland [92]
*Sycon* sp.	*Cladosporium* sp.	Helgoland, Germany [84]
unidentified species	*Cladosporium* sp.	Australia [27]
	*C. cladosporioides*, *Cladosporium* sp.	Antarctica [96]
	*C. colombiae*	Northeast Brazil [97]
	*Cladosporium* sp.	Manado, Indonesia [98]
	*Cladosporium* sp.	Xisha Islands, China [99]
	*C. cladosporioides*, *C. herbarum*, *C. ramotenellum*, *Cladosporium* sp.	Ria Aveiro, Portugal [35]
*Xestospongia* sp.	*C. halotolerans*	Andaman Sea, India [87]
Cnidaria
*Acropora formosa*	*Cladosporium* sp.	Great Barrier Reef, Australia [100]
*Acropora intermedia*	*Cladosporium* sp.	Hainan island, China [24]
*Acropora loripes*	*Cladosporium* sp.	Eilat, Israel [101]
*Acropora palmata*	*Cladosporium* sp.	Vera Cruz, Mexico [59]
*Anthogorgia ochracea*	*Cladosporium* sp.	Weizhou, China [102]
*Anthopleura xanthogrammica*	*C. allicinum*, *C. cladosporioides*, *C. colocasiae*, *C. halotolerans*, *C. sphaerospermum*, *C. tenuissimum*	Haizhou bay, China [103]
*Bathypathes* sp.	*C. ramotenellum*	North Atlantic Ocean [104]
*Cladiella krempfi*	*C. cladosporioides*, *C. dominicanum*, *C. sphaerospermum*	South China Sea [105]
corals	*C. cladosporioides*, *C. halotolerans*	Northeast Brazil [97]
	*Cladosporium* sp.	Hungyan island, China [106]
*Echinogorgia rebekka*	*Cladosporium* sp.	Weizhou, China [107]
*Gorgonia ventalina*	*C. sphaerospermum*, *Cladosporium* sp.	San Juan, Puerto Rico [108]
gorgonian	*C. sphaerospermum*	Eilat, Israel [87]
*Hydractinia echinata*	*C. sphaerospermum*	South Korea [109]
jellyfish	*C. oxysporum*	South Korea [110]
*Lepidisis* sp.	*C. cladosporioides*	North Atlantic Ocean [104]
*Leptogorgia obscura*	*C. dominicanum*	Manabí (Ecuador) [111]
*Leptogorgia* sp.	*C. sphaerospermum*	
*Montastrea annularis*	*C. sphaerospermum*	Barbados [112]
*Mussismilia hispida*	*Cladosporium* sp.	São Sebastiao, Brazil [113]
*Nemopilema nomurai*	*Cladosporium* sp.	Qingdao, China [114]
*Palythoa caribaeorum*	*C. cladosporioides*	São Sebastiao, Brazil [113]
		São Paulo, Brazil [115]
*Palythoa variabilis*	*Cladosporium* sp.	São Sebastiao, Brazil [113]
*Paragorgia* sp.	*C. sphaerospermum*	North Atlantic Ocean [104]
*Parantipathes* sp.	*C. ramotenellum*	
*Pelagia noctiluca*	*C. aggregatocicatricatum*, *C. allicinum*, *C. delicatulum*, *C. halotolerans*, *C. myrtacearum*, *C. westerdijkiae*	Giglio island, Italy [49]
*Porites lutea*	*Cladosporium* sp.	Kavaratti island, India [19]
	*C. halotolerans*	Weizhou islands, China [116]
*Porites* sp.	*C. sphaerospermum*	Rarotonga, Cook islands [112]
*Pseudosuberites andrewi*	*Cladosporium* sp.	Rameswaram, India [75]
*Sarcophyton tortuosum*	*C. halotolerans*, *C. sphaerospermum*	South China Sea [105]
scleractinian corals	*C. halotolerans*, *C. tenuissimum*, *Cladosporium* sp.	Hainan island, China [23]
soft coral	*Cladosporium* sp.	Guangzhou, China [117]
*Stylophora pistillata*	*C. halotolerans*	Gulf of Aqaba, Israel [22]
*Zoanthus solanderi*	*C. cladosporioides*	São Sebastiao, Brazil [113]
Bryozoa
*Hippodiplosia insculpta*	*C. sphaerospermum*	San Juan island, Washington [118]
unidentified species	*Cladosporium* sp.	Australia [27]
	*C. halotolerans*	Andaman Sea, India [87]
Platyhelminthes
*Trepaxonemata* sp.	*Cladosporium* sp.	Antarctica [119]
Nemertea
*Antarctonemertes valida*	*Cladosporium* sp.	Antarctica [119]
Anellida
*Sipunculus nudus*	*Cladosporium* sp.	Beihai, China [120]
*Urechis unicinctus*	*C. cladosporioides*, *Cladosporium* sp.	Yantai, China [120]
Mollusca
*Anadara broughtoni*	*C. sphaerospermum*	Sea of Japan, Russia [121]
*Cerastoderma edule*	*Cladosporium* sp.	Loire estuary, France [122]
*Eledone cirrhosa*	*C. sphaerospermum*	United Kingdom [20]
*Mizuhopecten yessoensis*	*C. cladosporioides*, *C. oxysporum*, *C. sphaerospermum*	Sea of Japan, Russia [123]
*Mytilus edulis*	*Cladosporium* sp.	Loire estuary, France [122]
*Mytilus galloprovincialis*	*Cladosporium* sp.	Oran, Algeria [124]
*Nacella concinna*	*C. halotolerans*, *Cladosporium* sp.	Antarctica [119]
*Nodipecten nodosus*	*Cladosporium* sp.	Angra dos Reis Bay, Brazil [125]
*Perna perna*	*Cladosporium* sp.	Guanabara bay, Brazil [126]
Arthropoda–crustacea
amphipod	*Cladosporium* sp.	King George island, Antarctica [78]
isopod	*C. halotolerans*	
*Portunus pelagicus*	*Cladosporium* sp.	Chanthaburi-Trat, Thailand [127]
*Portunus sanguinolentus*	*C. tenuissimum*	Taiwan [128]
*Xenograpsus testudinatus*	*C. cladosporioides*	Taiwan [129]
Echinodermata
*Apostichopus japonicus*	*C. brevicompactum*, *C. oxysporum*, *C. sphaerospermum*	Sea of Japan, Russia [21]
*Cucumaria japonica*	*C. brevicompactum*, *C. sphaerospermum*	
*Eupentacta fraudatrix*	*C. brevicompactum*, *C. oxysporum*, *C. sphaerospermum*	
*Holothuria leucospilota*	*C. halotolerans*	Pangkor Island, Malaysia [130]
*Holothuria poli*	*C. sphaerospermum*	Tabarka peninsula, Tunisia [131]
Chordata–tunicata
*Cystodytes dellechiajei*	*Cladosporium* sp.	L'Escala, Spain [132]
*Didemnum fulgens*	*Cladosporium* sp.	
*Didemnum maculosum*	*Cladosporium* sp.	Almuñecar, Spain [87]
*Didemnum* sp.	*Cladosporium* sp.	São Sebastiao, Brazil [82]
*Pycnoclavella communis*	*Cladosporium* sp.	L'Escala, Spain [132]
*Salpa* sp.	*Cladosporium* sp.	King George island, Antarctica [78]
Chordata–pisces
*Arctoscopus japonicus* (eggs)	*C. bruhnei*, *C. cladosporioides*, *C. fusiforme*, *C. halotolerans*, *C. perangustum*, *C. pseudocladosporioides*, *C. rectoides*, *C. ramotenellum*, *C. sphaerospermum*, *C. xylophilum*	South Korea [133]
*Cromileptis altivelis*	*C. cladosporioides*	Australia [134]
*Lates calcarifer*	*C. halotolerans*	Daya bay, China [135]
*Lutjanus campechanus*	*C. sphaerospermum*	Horn island, Mississippi [136]
*Trachinotus blochii*	*C. halotolerans*	Daya bay, China [135]
unidentified species	*C. sphaerospermum*	Maizuru bay, Japan [137]
Chordata–reptilia*
*Caretta caretta*	*C. cladosporioides*	Mostardas beach, Brazil [15]
	*Cladosporium* sp.	Apulia, Italy [138]
*Chelonia mydas*	*Cladosporium* sp.	Heron reef, Australia [139]
	*C. anthropophilum*	Santos basin, Brazil [16]
	*Cladosporium* sp.	Indian River lagoon, Florida [17]
*Eretmochelys imbricata*	*C. cladosporioides*	Puerto Morelos reef, Mexico [18]
Chordata–mammalia
*Hydrurga leptonyx*	*C. halotolerans*	Primavera Cape, Antarctica [140]
*Lobodon carcinophagus*	*C. halotolerans*	
*Orcinus orca*	*Cladosporium* sp.	Salish sea, Canada [141]
*Phocoena* sp.	*Cladosporium* sp.	Black sea [142]
*Stenella coeruleoalba*	*Cladosporium* sp.	Italian coast [143]
*Tursiops truncatus*	*Cladosporium* sp.	Black sea [142]
	*C. halotolerans*	Gulf of Mexico [144]

**Figure 1.**
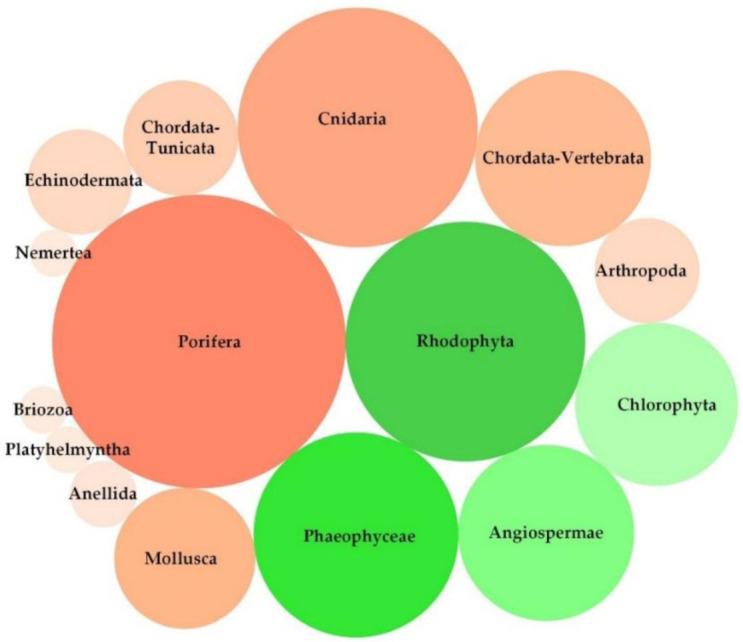
Bubble chart representing the number of findings of *Cladosporium* in association with marine plant (green) and animal (orange) organisms.

The prevalence of the findings of symbiotic *Cladosporium* with reference to the plant and animal phyla is represented in the bubble chart shown in Figure 1.

## 4. Secondary metabolites: Chemical structures

**Figure 2.**
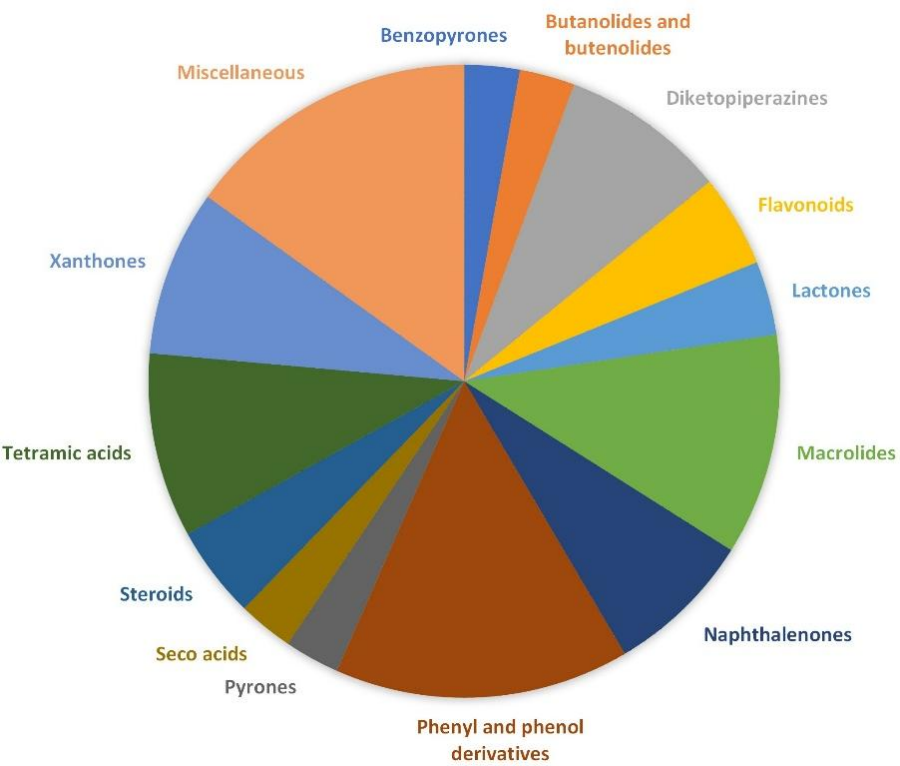
Pie chart representing the distribution of chemical classes of secondary metabolites produced by fungi of the genus *Cladosporium* recovered from marine organisms.

From our revision of the available literature, as many as 106 compounds were reported as secondary metabolites of *Cladosporium* strains associated with marine organisms so far (Table 3); a good number of them (29) were first identified from this biological source. These metabolites belong to different classes of natural products (Figure 2), such as benzopyrones, diketopiperazines, flavonoids, lactones, macrolides, naphthalenones, pyrones, tetramic acids, and steroids, revealing a great biosynthetic aptitude of these fungi.

**Table 3.** Secondary metabolites identified in axenic cultures of *Cladosporium* isolates from marine organisms. The names of novel compounds are underlined.

Code	Compound	Structure	References
Benzopyrones	
1	Coniochaetone A	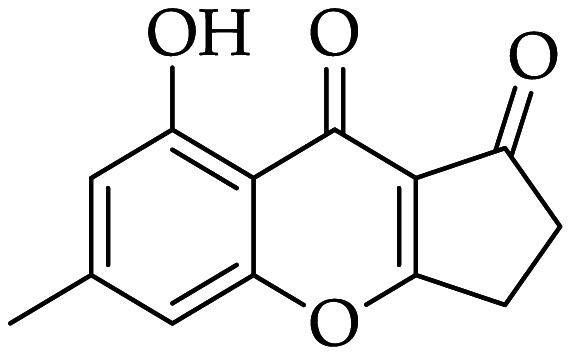	[116]
2	Coniochaetone B	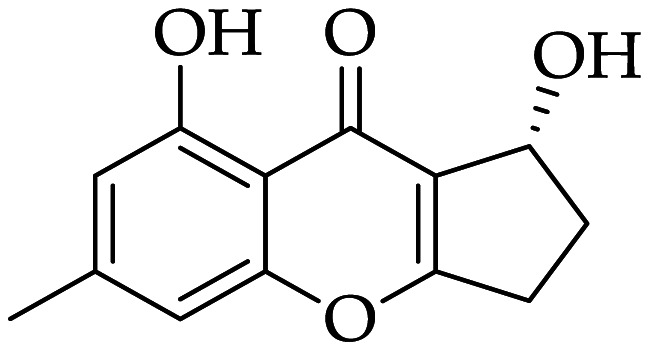	[94],[116]
3	Coniochaetone K	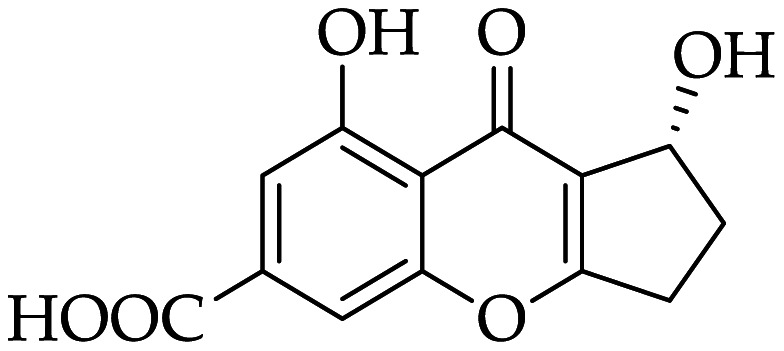	[94],[116]
Butanolides and butenolides	
4	Cladospolide F	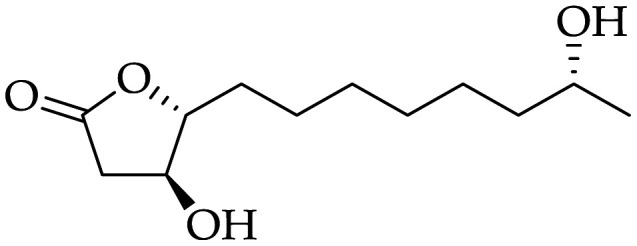	[117]
5	*iso*-Cladospolide B	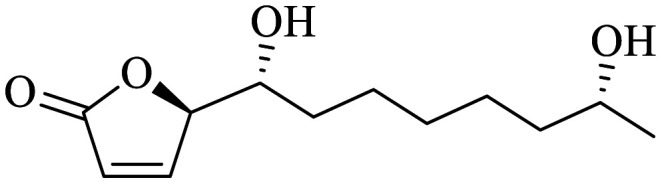	[86],[94],[95],[102],[117]
6	11-Hydroxy-*γ*-dodecalactone	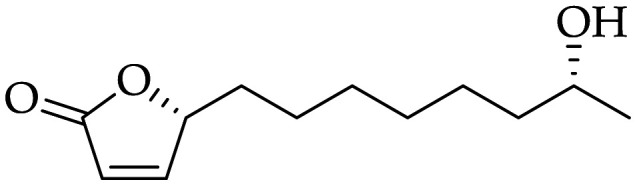	[117]
Diketopiperazines	
7	Cyclo (Leu, Ile)	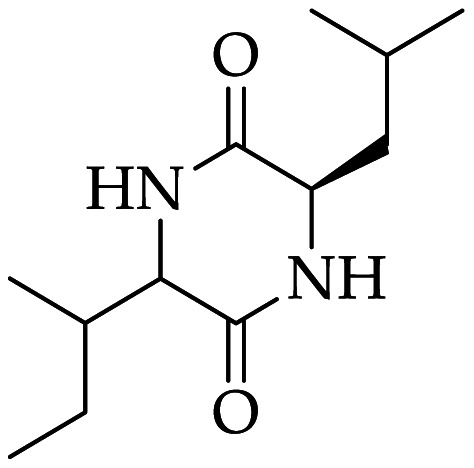	[110]
8	Cyclo (Leu, Leu)	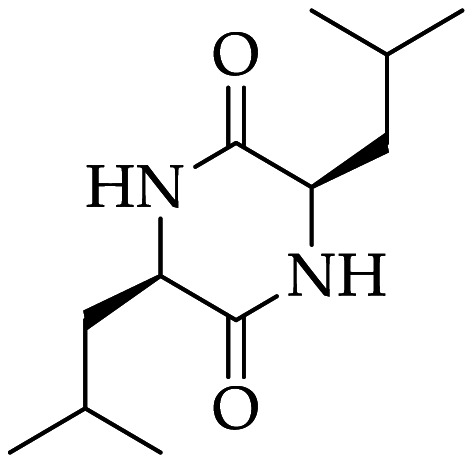	[110]
9	Cyclo (Pro, Val)	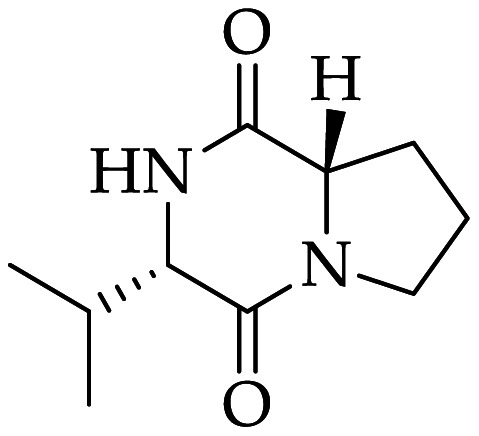	[72]
10	Cyclo (Trp, Pro)	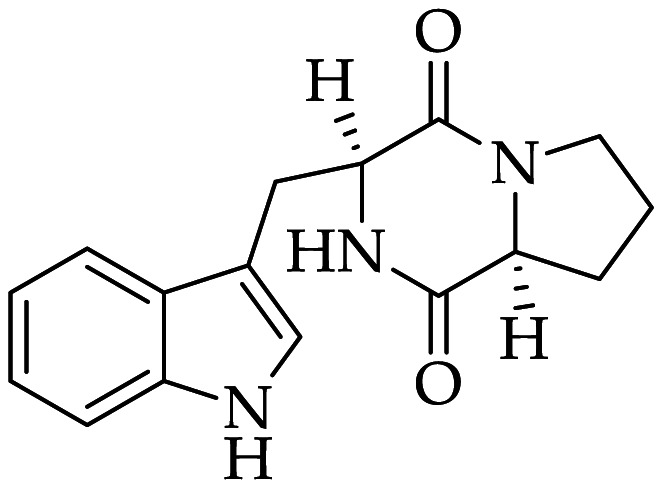	[72]
11	Cyclo (Phe, Ile)	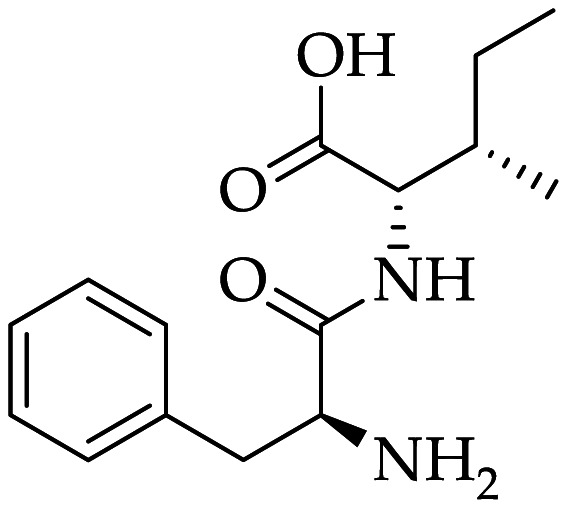	[110]
12	Cyclo (Phe, Leu)	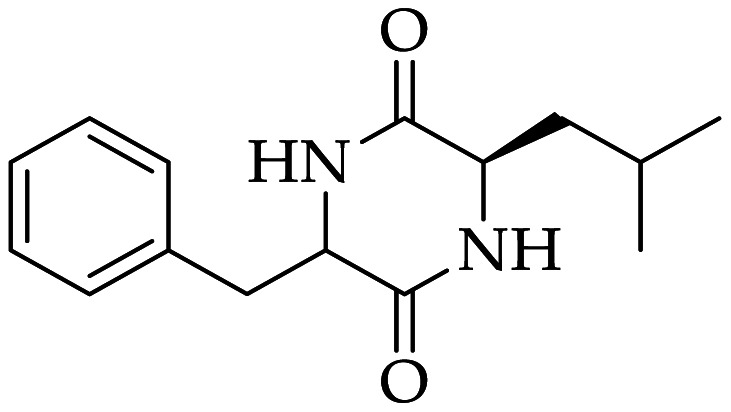	[110]
13	Cyclo (Phe, Phe)	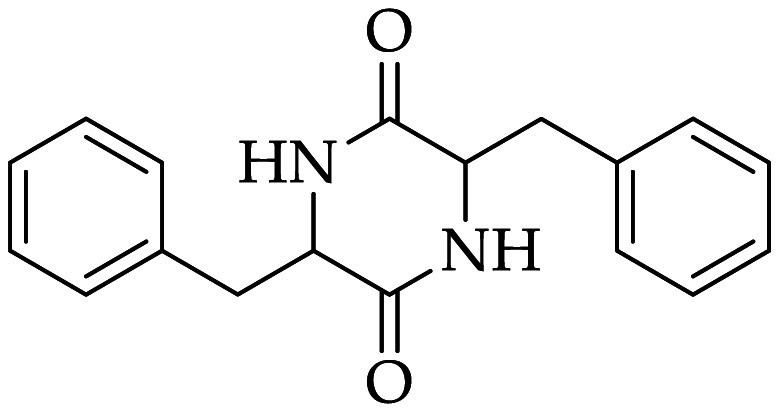	[110]
14	Cyclo (Phe, Pro)	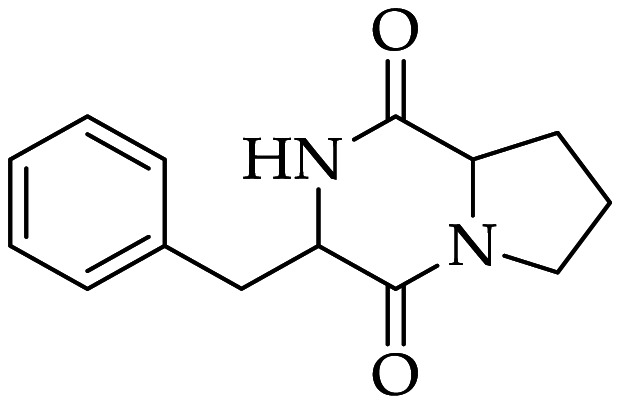	[94]
15	Cyclo (Phe, Val)	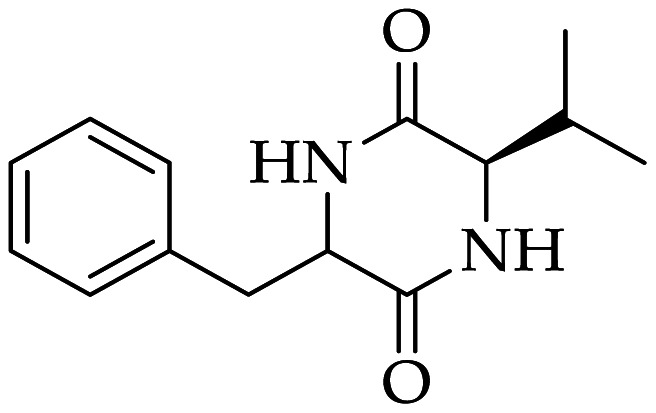	[110]
Flavonoids	
16	Daidzein	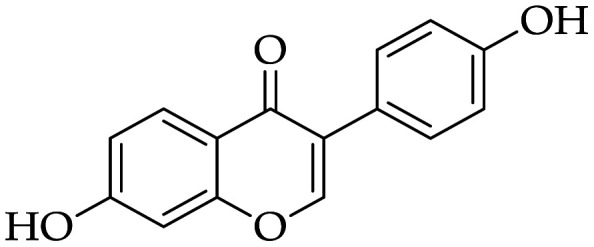	[110]
17	Genistein	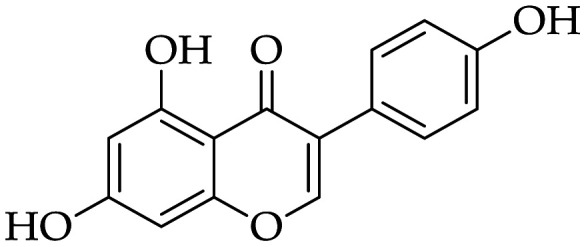	[110]
18	Glycitein	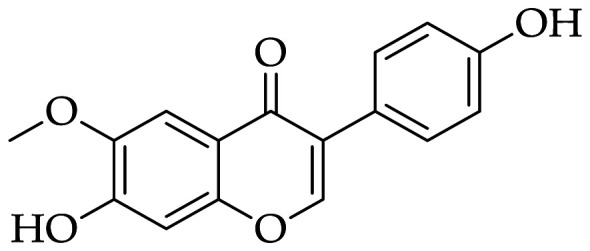	[110]
19	(2*S*)-7,4′-Dihydroxy-5-methoxy-8-(*γ*,*γ*-dimethylallyl)-flavanone	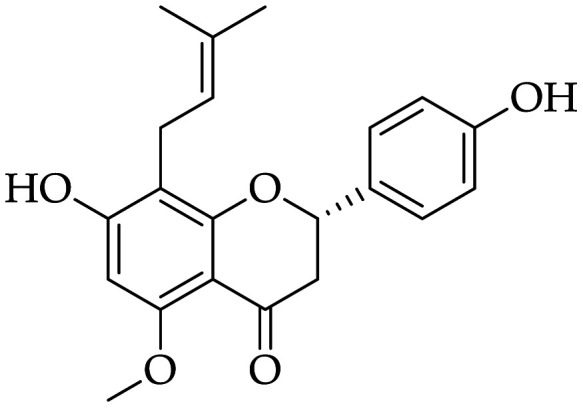	[98]
20	Prunetin	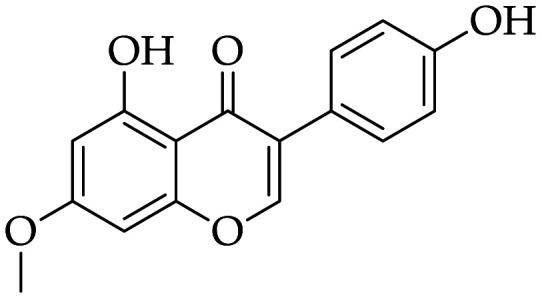	[110]
Lactones	
21	Cladosporactone A	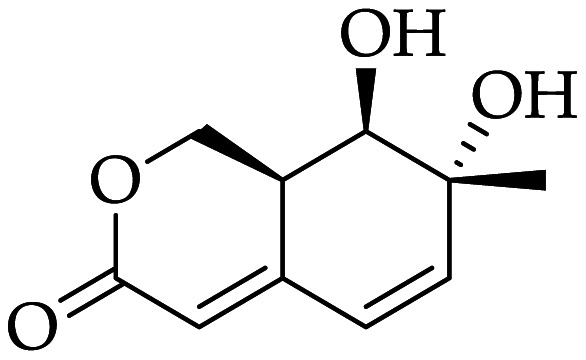	[94]
22	Cladosporamide A	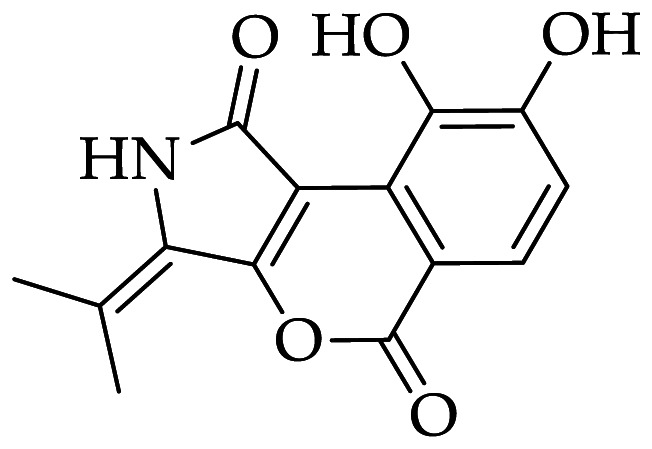	[98]
23	Herbaric acid	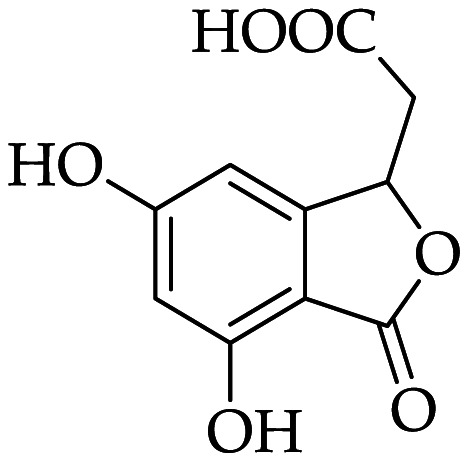	[83]
24	(*R*)-Mevalonolactone	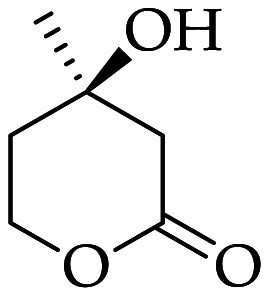	[72]
Macrolides	
25	Cladospolide B	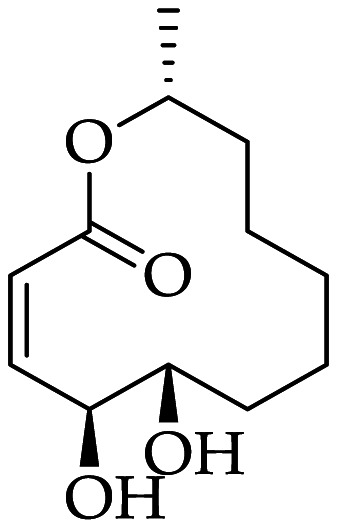	[86],[102]
26	Dendrodolide A	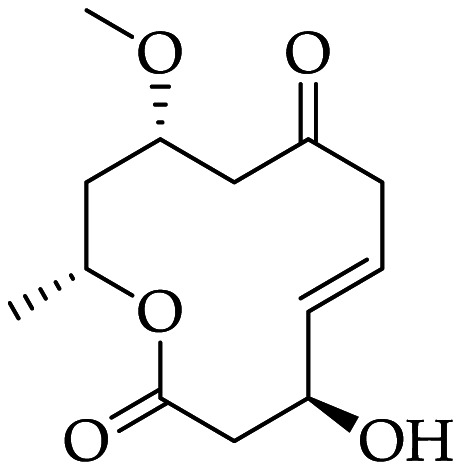	[102]
27	Dendrodolide C	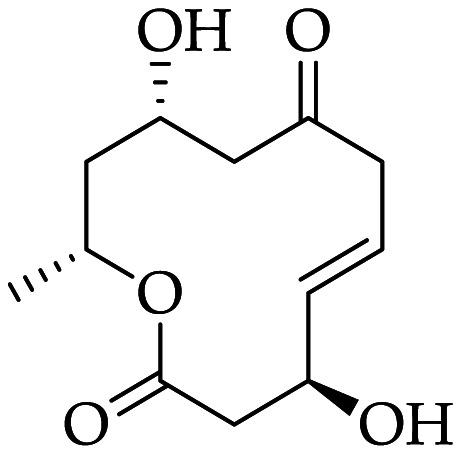	[102]
28	Dendrodolide M	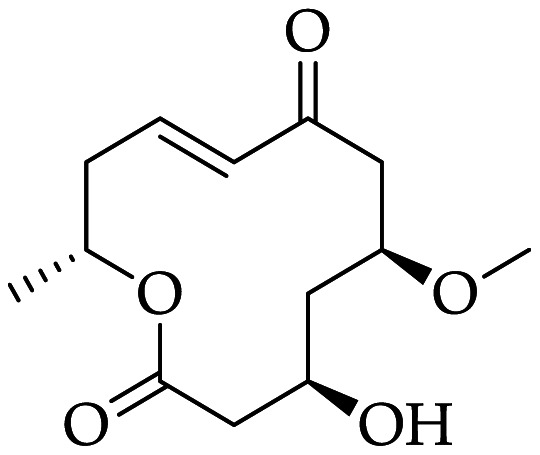	[102]
29	Dendrodolide L	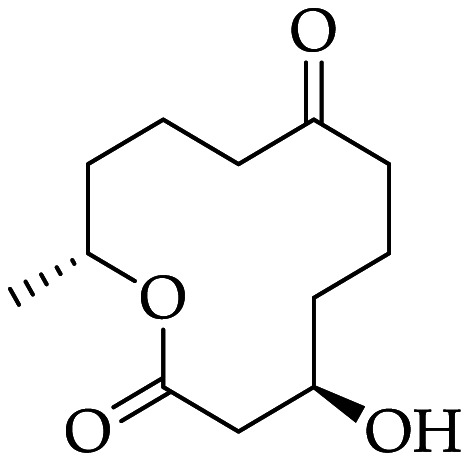	[102]
30	Pandangolide 1	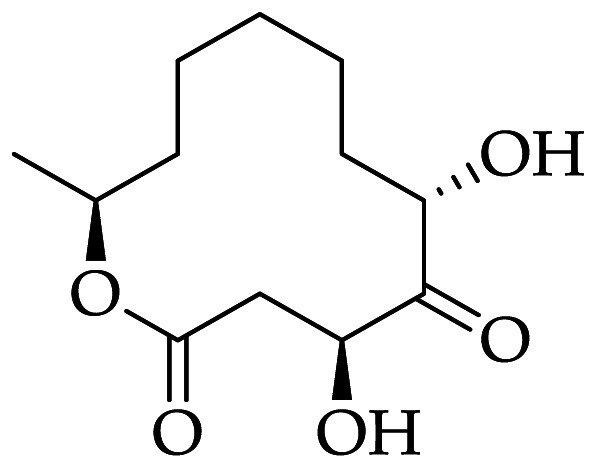	[95]
31	Pandangolide 1a	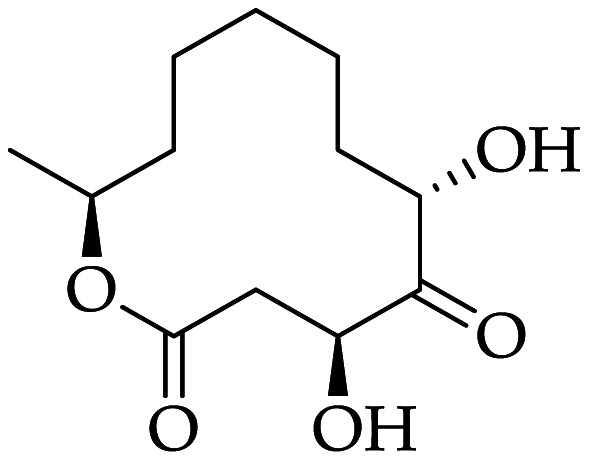	[95]
32	Pandangolide 2	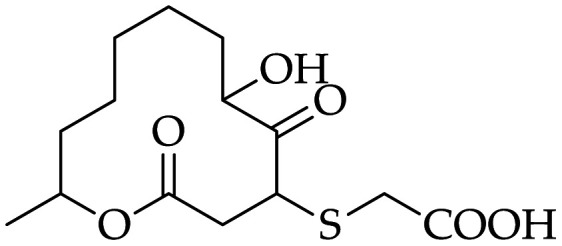	[86]
33	Pandangolide 3	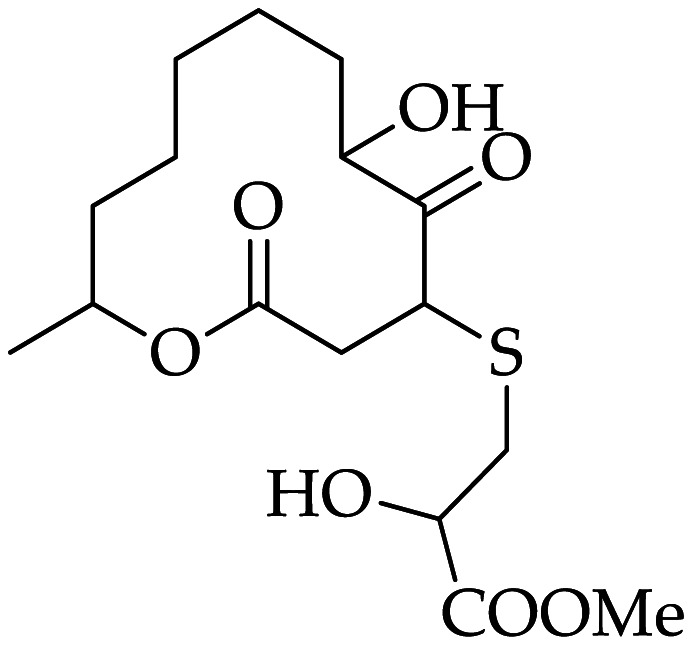	[86]
34	Pandangolide 4	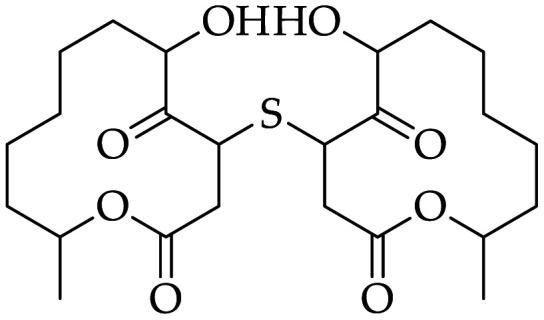	[86]
35	Sporiolide A	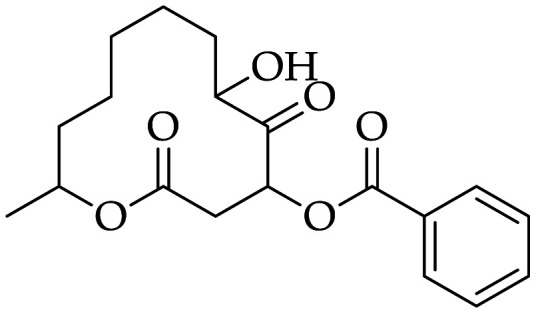	[67]
36	Sporiolide B	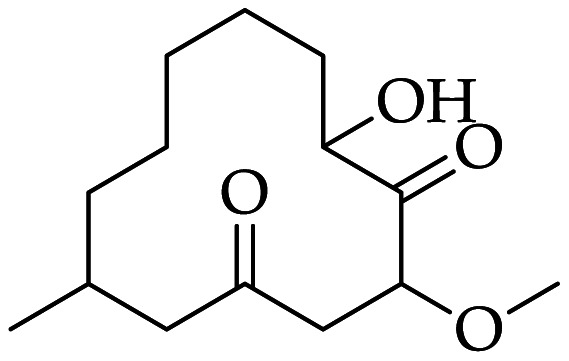	[67]
Naphthalenones	
37	Altertoxin XII	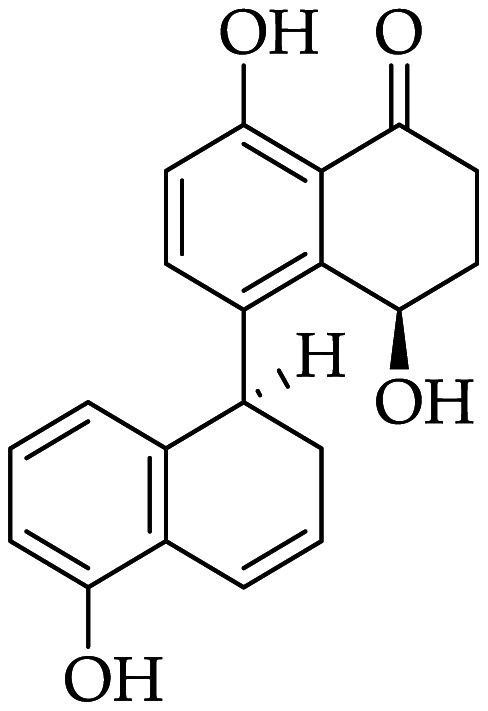	[94]
38	Cladosporol C	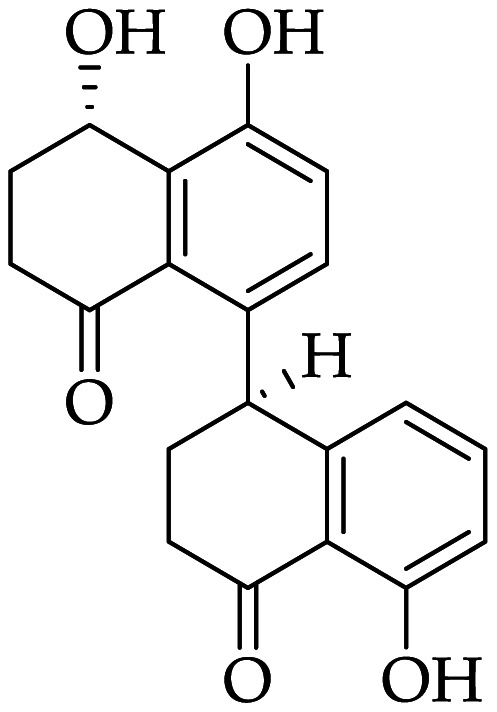	[71]
39	Cladosporol F	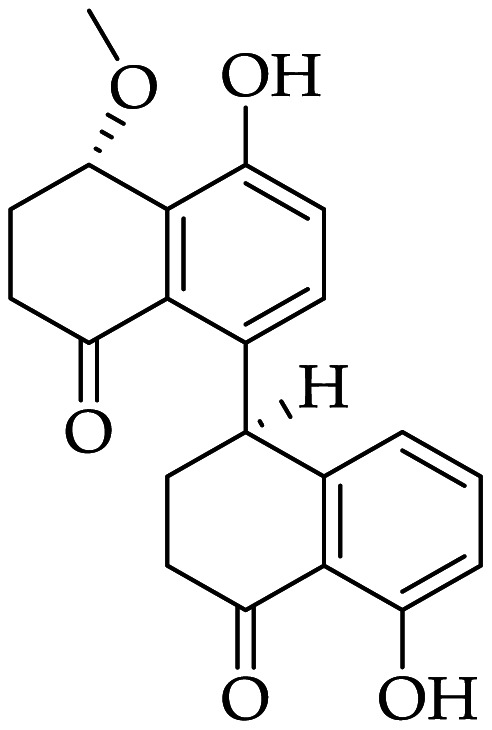	[71],[94]
40	Cladosporol G *bis*	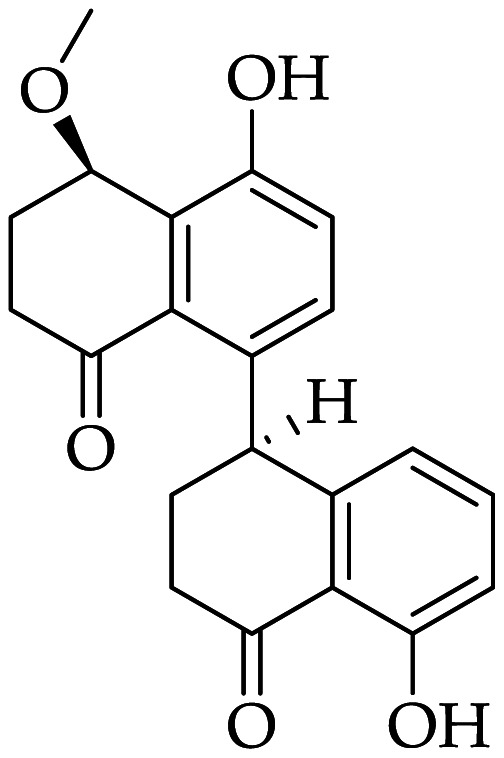	[71]
41	Cladosporol H	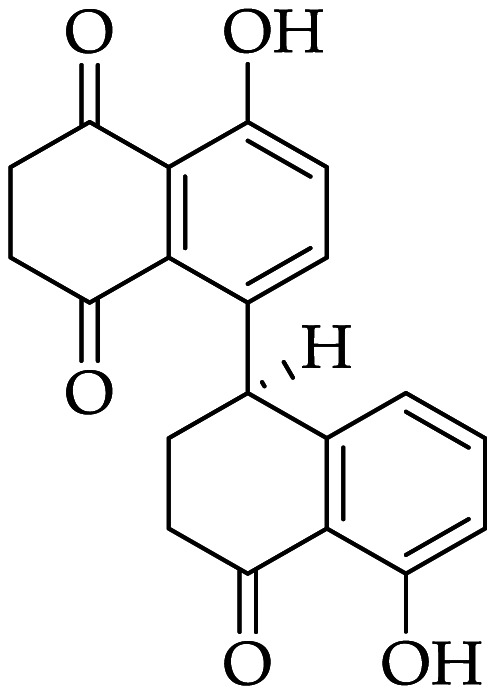	[71],[94]
42	Cladosporol I	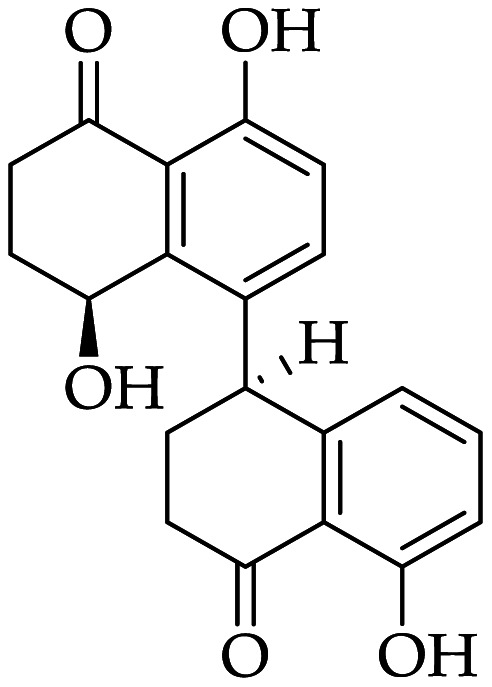	[71]
43	Cladosporol J	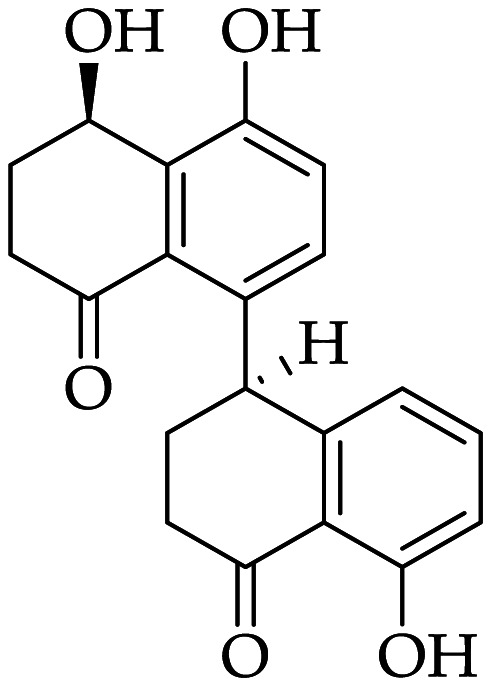	[71]
44	4,8-Dihydroxy-1-tetralone	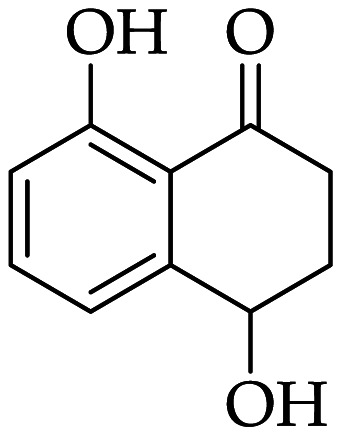	[94]
Phenyl and phenol derivatives	
45	*N*-Acetylamicoumacin C	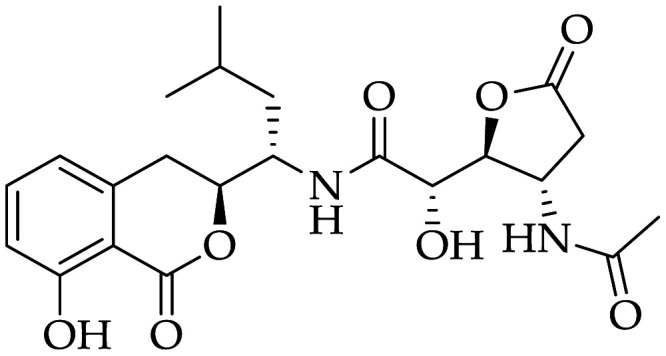	[88]
46	α-Acetylorcinol	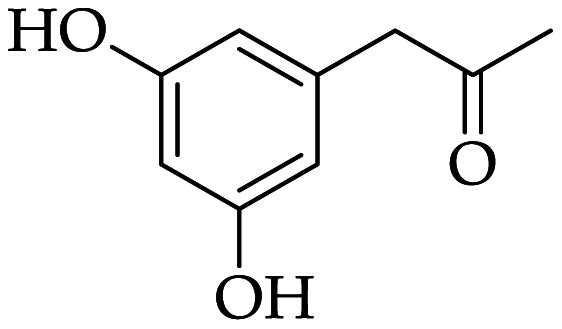	[94]
47	*N*-acetyl-tyramine	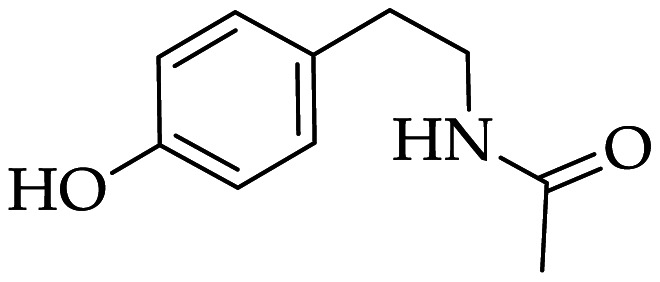	[72]
48	Altertoxin IX	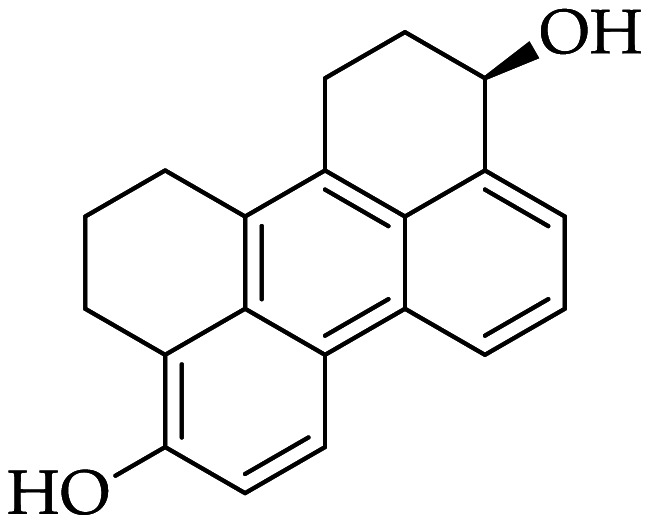	[94]
49	Cladosporin C	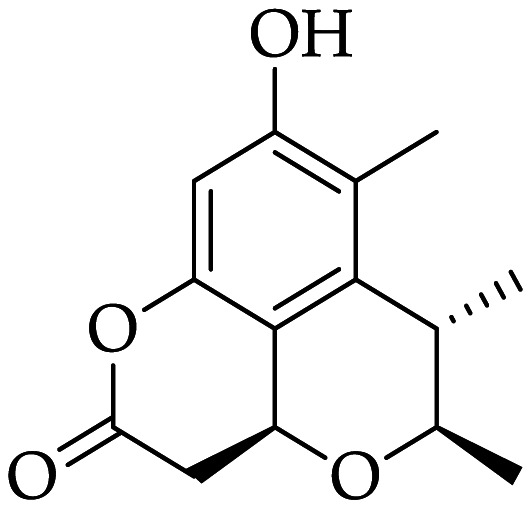	[94]
50	1-(3,5-Dihydroxy-4-methylphenyl) propan-2-one	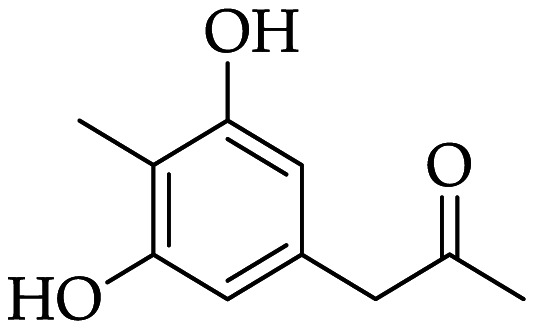	[94]
51	*p*-Hydroxy benzoic acid methyl ester	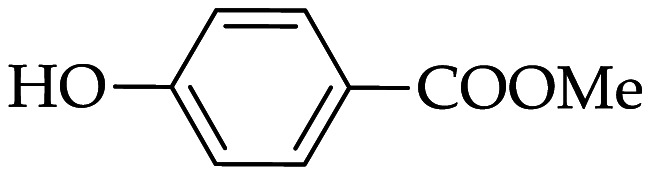	[72]
52	*p*-Hydroxybenzyl alcohol	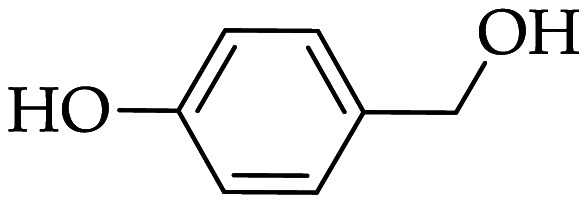	[72]
53	1-(4-Hydroxy-3,5-dimethoxyphenyl)-1,2-ethanediol	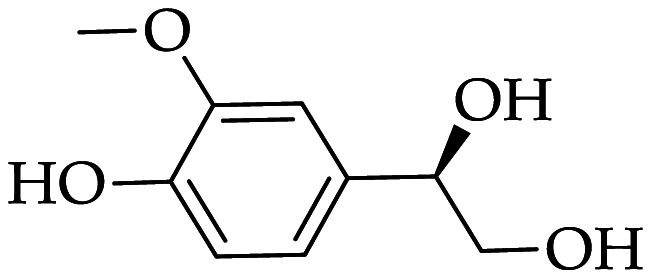	[110]
54	*p*-Hydroxyphenylacetic acid	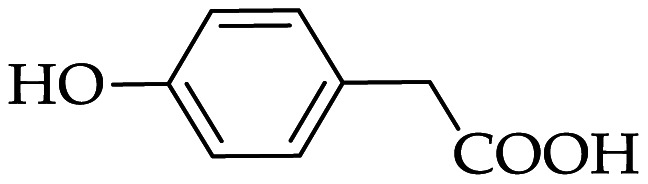	[72]
55	(2*R*,3*S*)-1-(4-Hydroxyphenyl) butane-2,3-diol	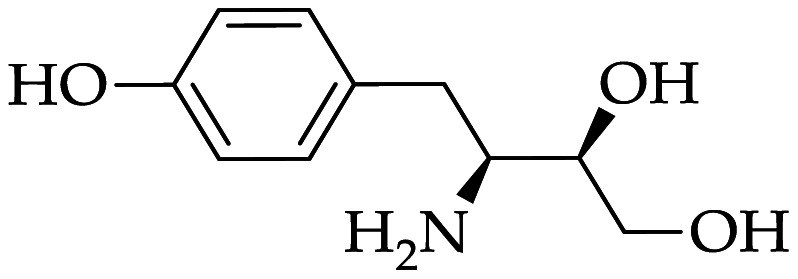	[88]
56	*p*-Hydroxyphenylethyl alcohol	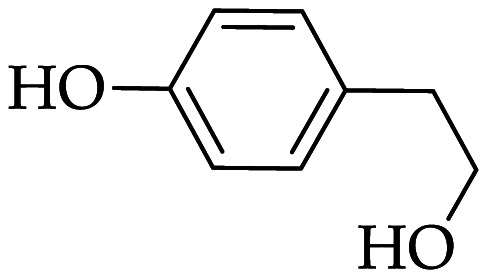	[72]
57	*N*-(2-phenylethyl) acetamide	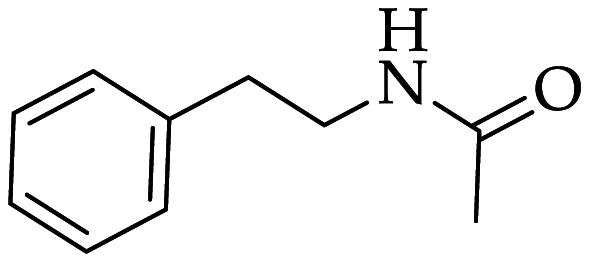	[88]
58	Phenylacetic acid	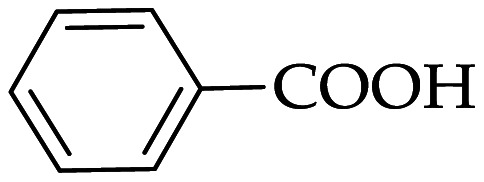	[72]
59	l-*β*-Phenyllactic acid	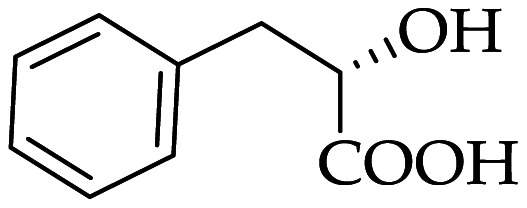	[72]
60	*α*-Resorcylic acid	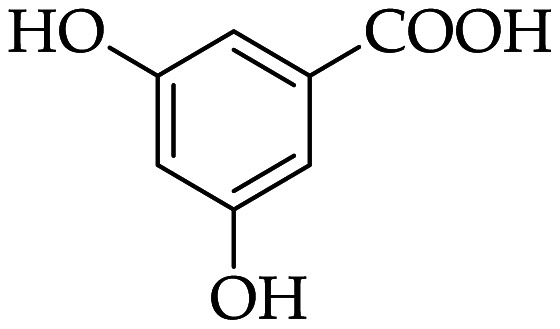	[72]
Pyrones	
61	Citreoviridin A	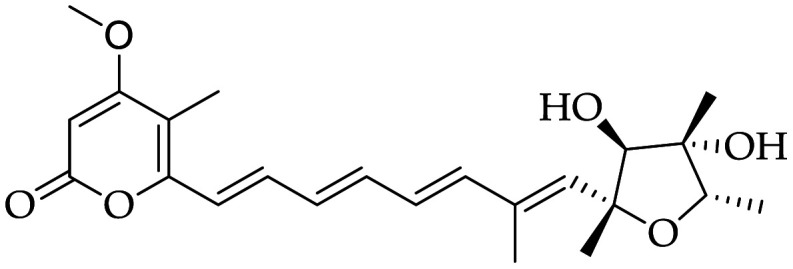	[83]
62	Herbarin A	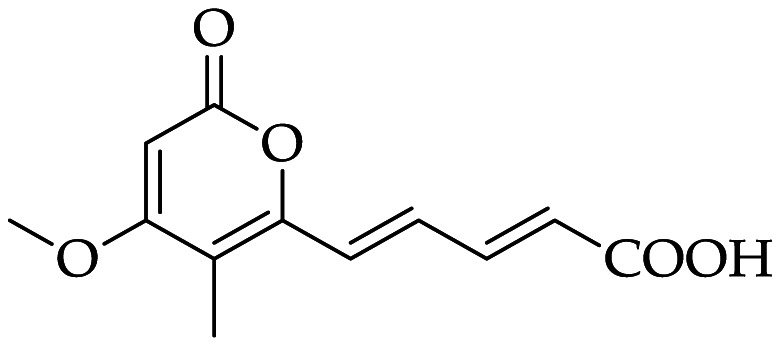	[83],[94]
63	Herbarin B	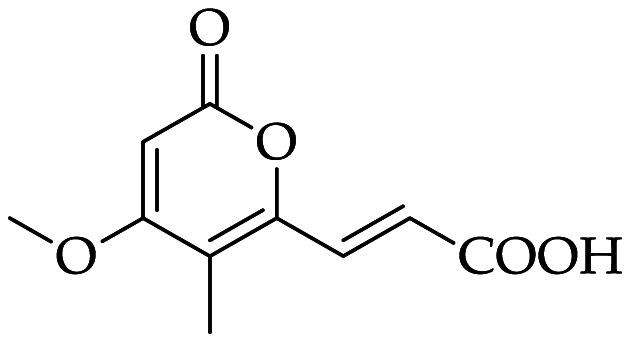	[83],[94]
Seco acids	
64	Cladospolide E	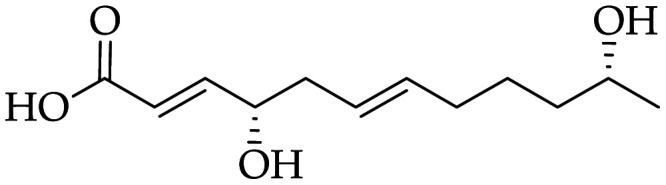	[117]
65	*seco*-Patulolide A	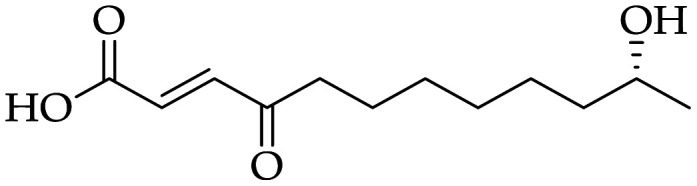	[117]
66	*seco*-Patulolide C	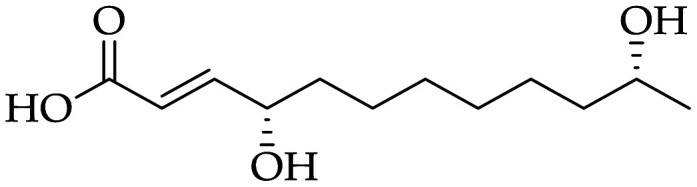	[117]
Steroids	
67	3α-Hydroxy-pregn-7-ene-6,20-dione	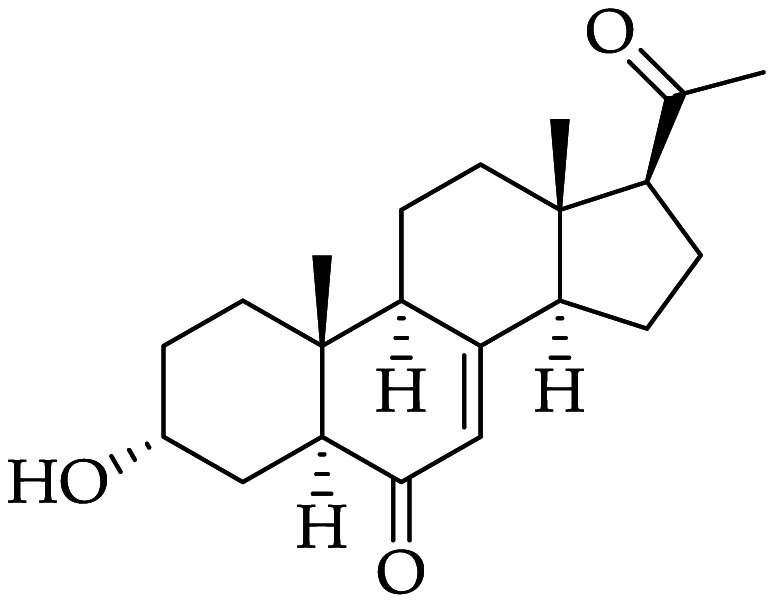	[145]
68	Peroxyergosterol (=(22*E*)-5α,8α-epidioxyergosta-6,22-dien-3*β*-ol)	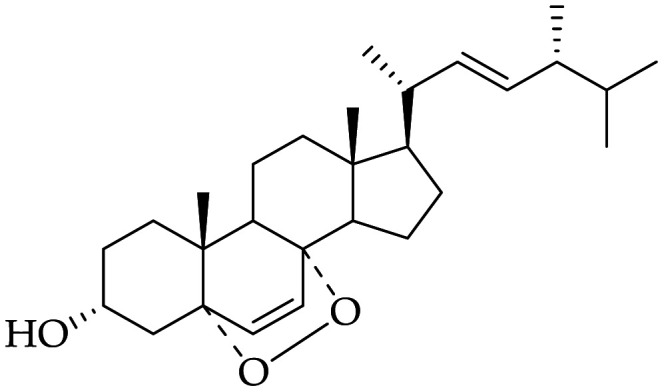	[89]
69	Cladopsol E	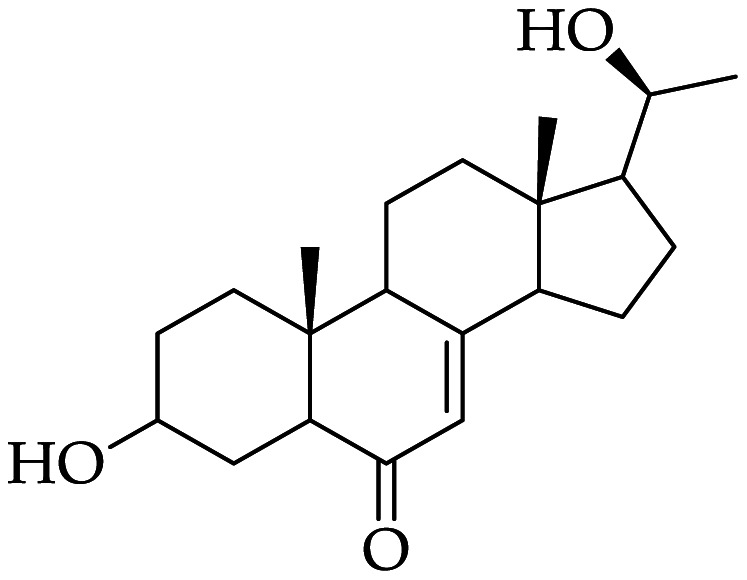	[110]
70	Cladosporisteroid B	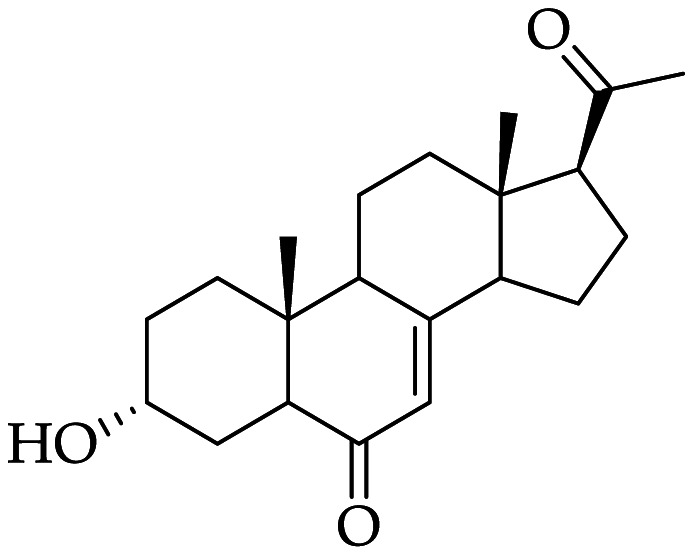	[110]
71	Olean-12-ene-3β,22α,24-triol	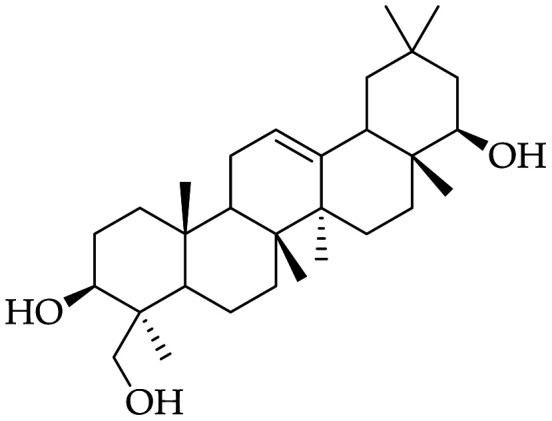	[110]
Tetramic acids	
72	Cladodionen	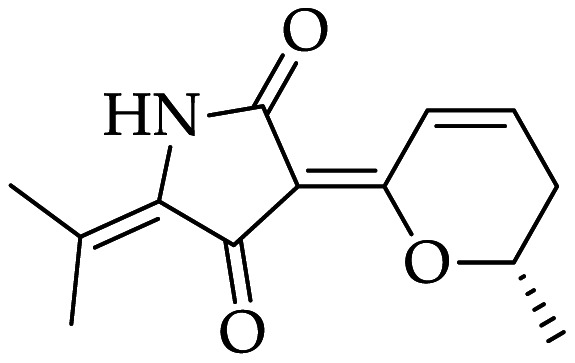	[99]
73	Cladosin B	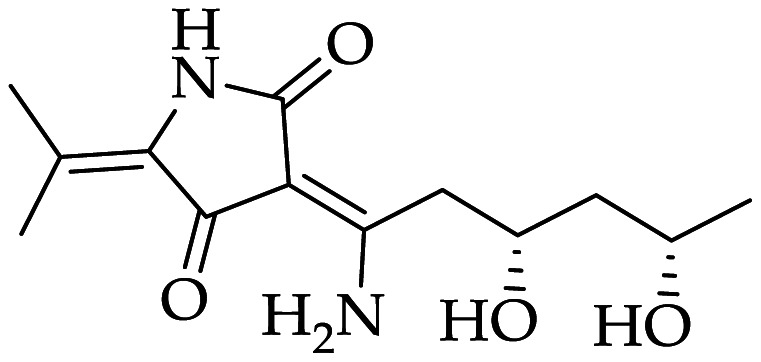	[146]
74	Cladosin C	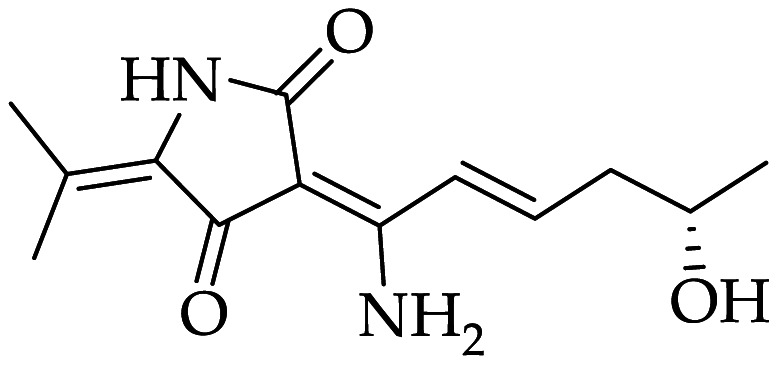	[146]
75	Cladosin F	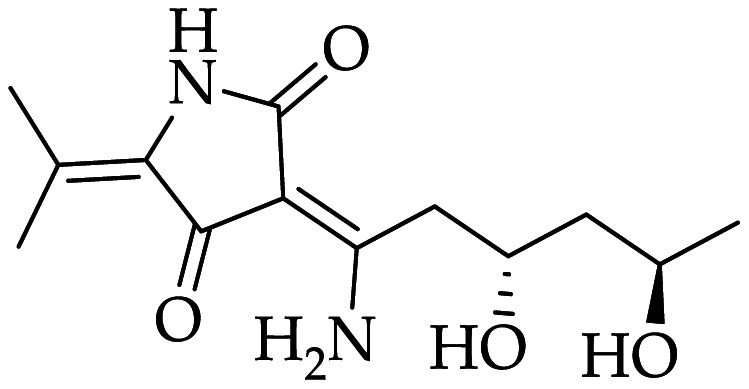	[146]
76	Cladosin L	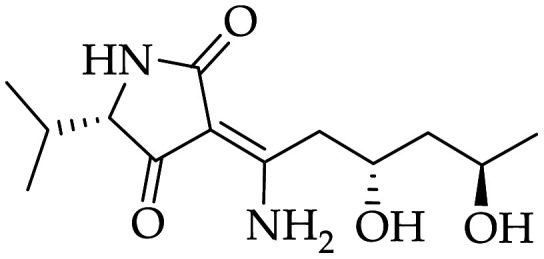	[146]
77	Cladosporicin A	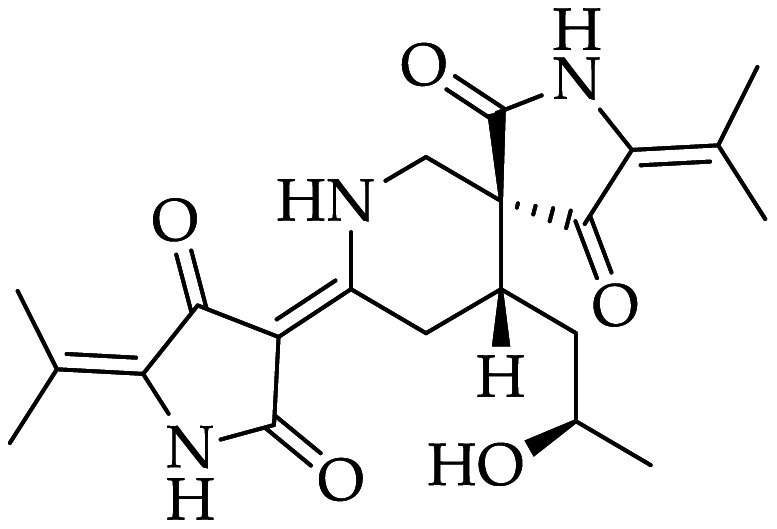	[109]
78	Cladosporiumin H	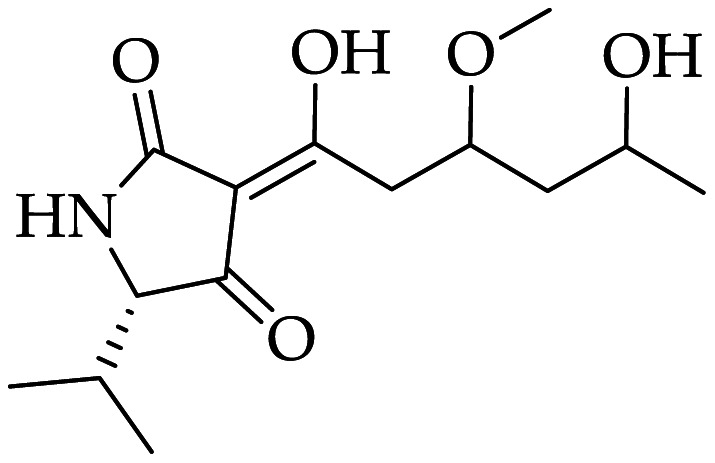	[94]
79	Cladosporiumin I	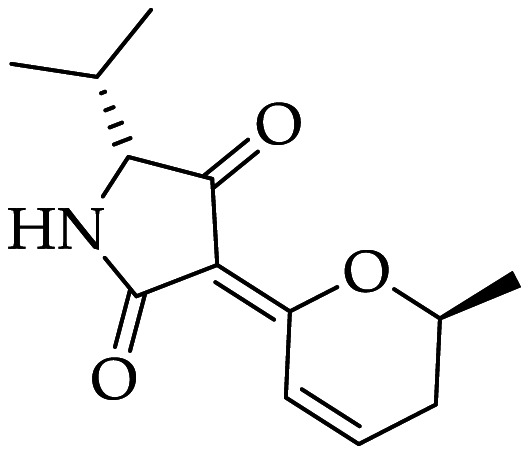	[94]
80	Cladosporiumin I *bis*	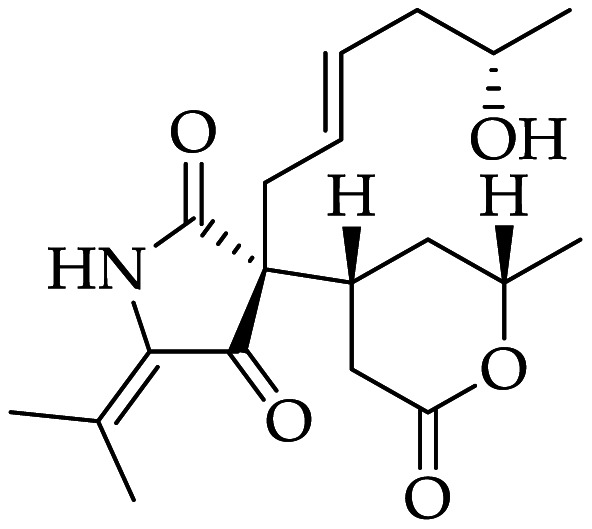	[109]
81	Cladosporiumin J *bis*	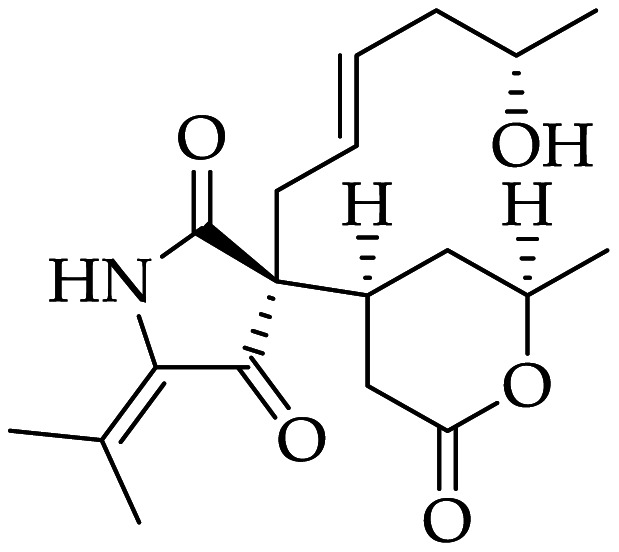	[109]
Xanthones	
82	Conioxanthone A	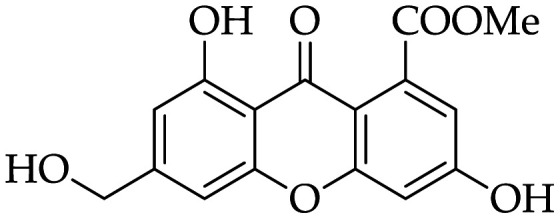	[94],[116]
83	3,8-Dihydroxy-6-methyl-9-oxo-9*H*-xanthene-1-carboxylate	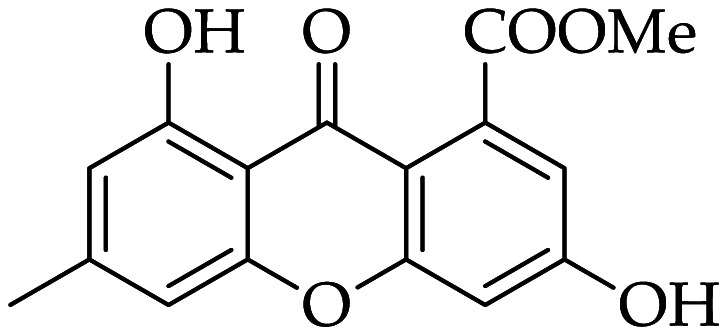	[116]
84	*α*-Diversonolic ester	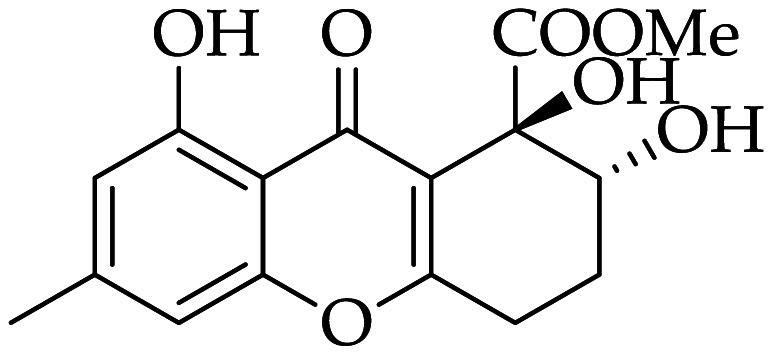	[94],[116]
85	*β*-Diversonolic ester	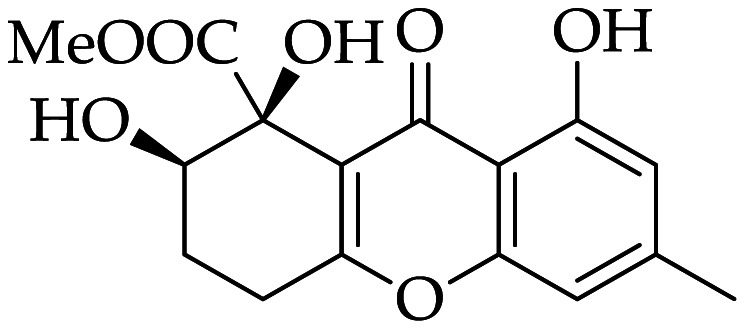	[116]
86	8-Hydroxy-6-methylxanthone-1-carboxylic acid	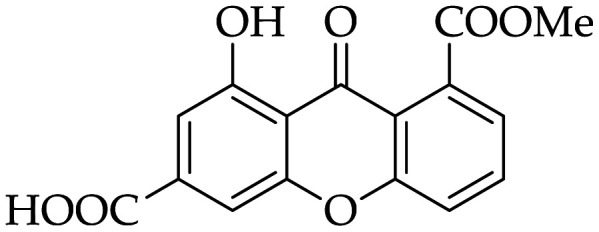	[116]
87	8-(Methoxycarbonyl)-1-hydroxy-9-oxo-9*H*-xanthene-3-carboxylic acid	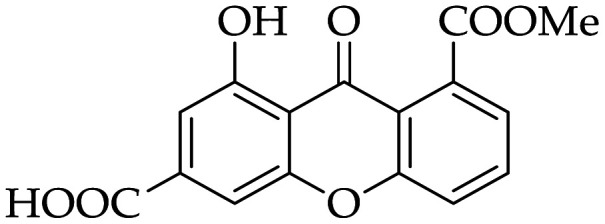	[116]
88	Methyl 8-hydroxy-6-(hydroxymethyl)-9-oxo-9*H*-xanthene-1-carboxylate	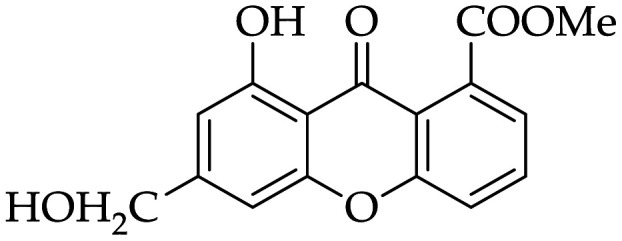	[94],[116]
89	Methyl 8-hydroxy-6-methyl-9-oxo-9*H*-xanthene-1-carboxylate	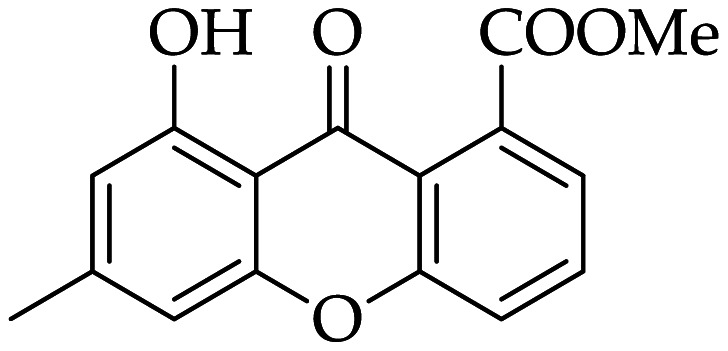	[94],[116]
90	Vertixanthone	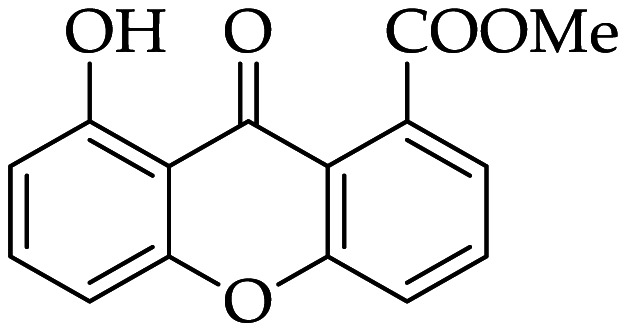	[94],[116]
Miscellaneous	
91	Acetyl Sumiki's acid	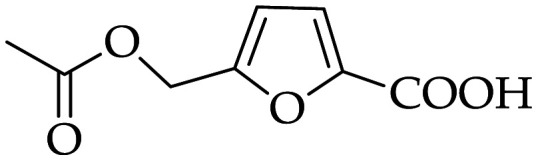	[86]
92	*N*-*β*-acetyltryptamine	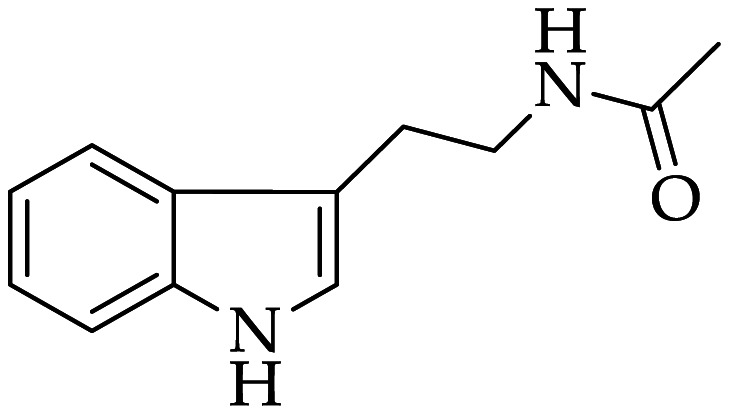	[72]
93	Cladosacid	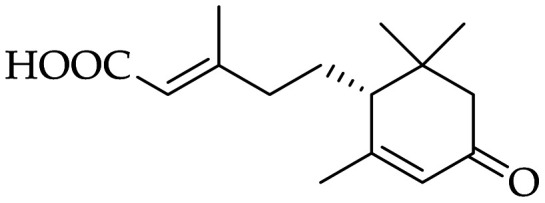	[99]
94	Cladopsol A	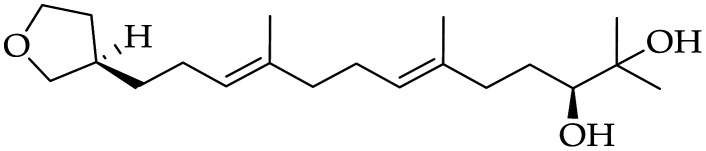	[110]
95	Cladopsol B	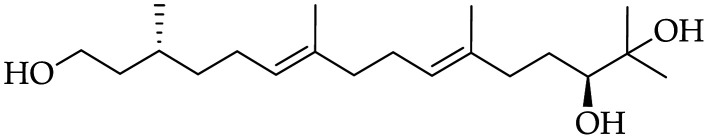	[110]
96	Cladopsol C	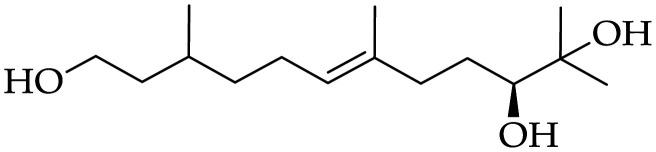	[110]
97	Cladopsol D	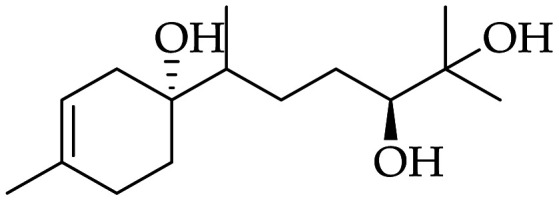	[110]
98	Cladosporilactam A	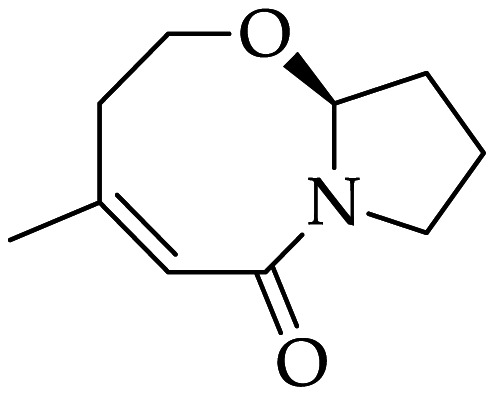	[102]
99	3,4-Dihydroxy-β-bisabolol	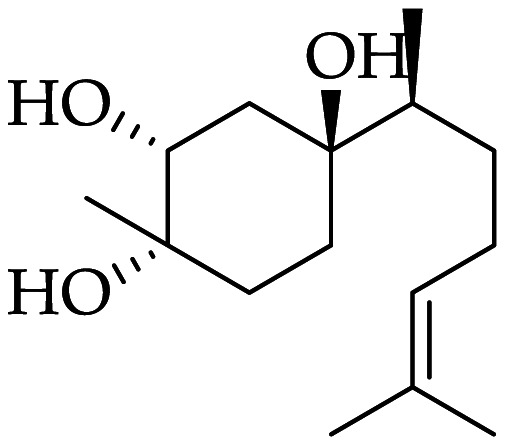	[110]
100	*N*-hydroxy-*N*-(2-(1-hydroxy-2-methoxy-1*H*-indol-3-yl) ethyl acetamide	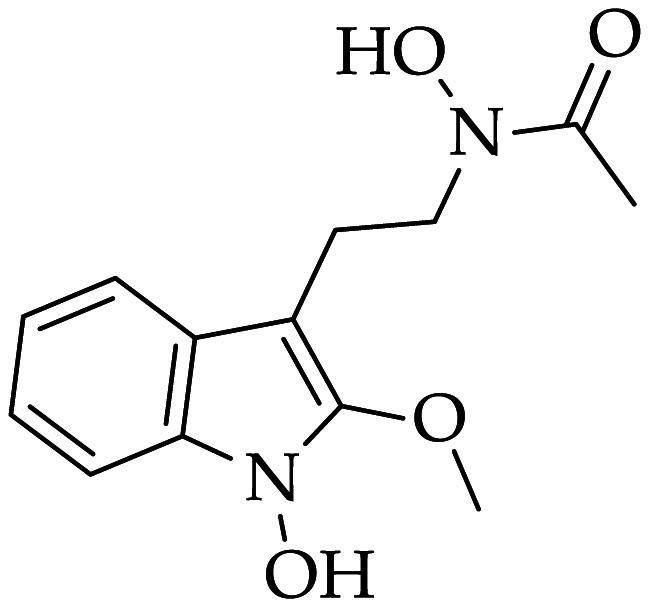	[88]
101	*N*-(2-(1*H*-Indol-3-yl) ethylacetamide	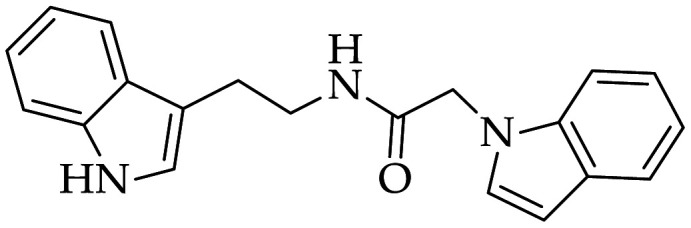	[88]
102	Malettinin B	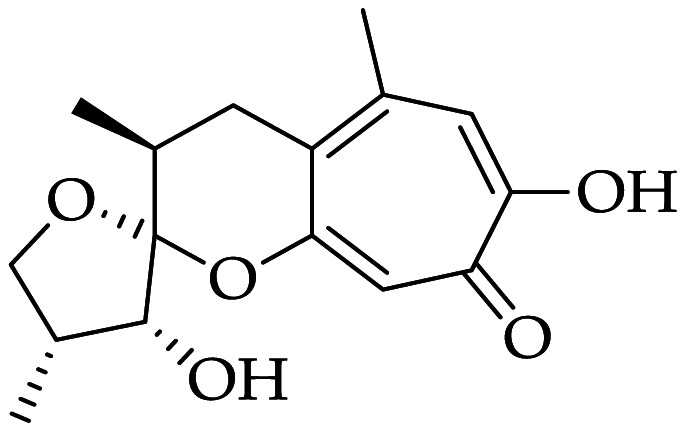	[94]
103	*N*-methyl-1*H*-indole-2-carboxamide	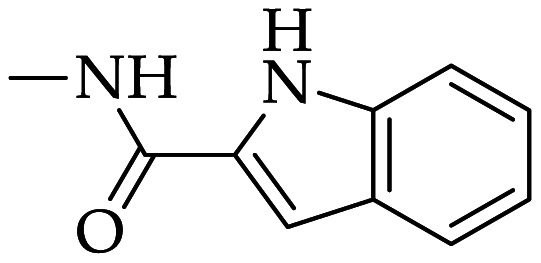	[147]
104	Nicotinic acid	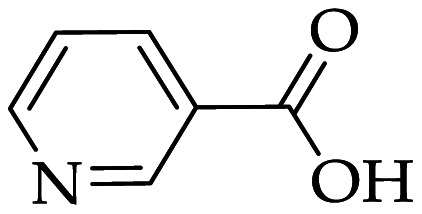	[72]
105	Sumiki's acid	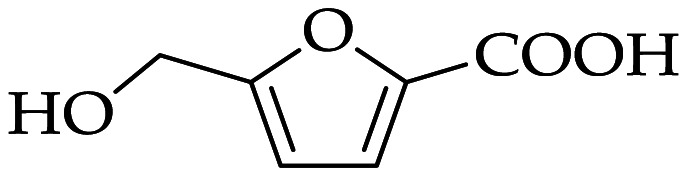	[86],[94]
106	Tremulenediol A	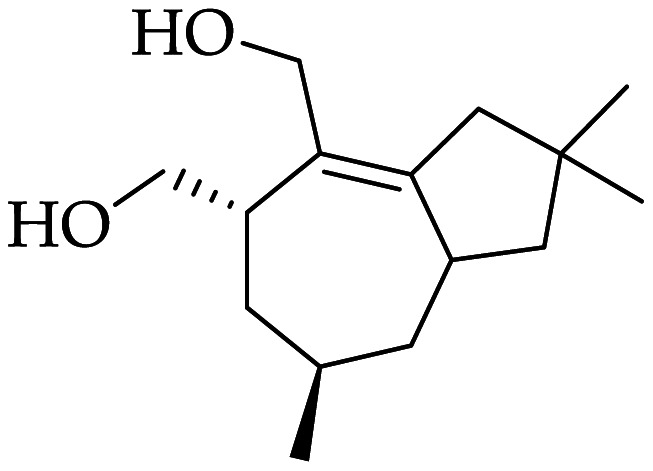	[110]

Some strains were particularly prolific in terms of metabolite production. This is the case with *C. halotolerans* GXIMD 02502, which yields twelve benzopyrone and xanthone derivatives, including a new compound with an uncommon carboxyl group at C-8 named coniochaetone K [116]. This product continues the series of coniochaetones, which are characterized by an unusual cyclopentabenzopyran-4-one skeleton [148]. Even if benzopyrone derivatives are well known as metabolites of fungi and plants [149],[150], members of this class containing fused cyclopentanoid rings are rare. From a comparison of the structural characteristics of coniochaetones, it can be inferred that the carboxyl in coniochaetone K and the carbonyl group at C-1 in coniochaetone A promote stronger cytotoxic activity [116].

Another example is represented by a jellyfish-derived strain of *C. oxysporum*, which was found to produce five undescribed compounds; namely two acyclic diterpenes (cladopsols A-B), two sesquiterpenes (cladopsols C-D), and the C21-ecdysteroid cladopsol E, along with 15 known compounds belonging to various classes, such as diketopiperazines, flavonoids, and phenyl derivatives [110].

Cladosporols are naphthalenones representing unique metabolites of the genus *Cladosporium*, which have attracted attention for their valuable biological properties [151]. In addition to the known cladosporol C, the algal-derived endophytic strain *C. cladosporioides* EN-399 produces four new derivatives (cladosporols F–J), which broaden the pattern of these products in view of further assessments of their bioactivity [71]. The work carried out for the structural elucidation of the new compounds also brought to the revision of the absolute configurations of the known cladosporols from (4′*R*) to (4′*S*).

Four new tetramic acids, namely cladosporicin A, cladosporiumins I *bis* and J *bis*, and cladosin L, were isolated for the first time from the hydroid-derived strain SW67 of *C. sphaerospermum* [109],[146]. Cladosporicin A contains an unprecedented 2,7-diazaspiro[4.5]decane-1,4-dione skeleton conjugated with a 2,4-pyrrolidinedione moiety, while the two cladosporiumins are stereoisomers presenting a tetramic acid scaffold with a quaternary (C-3) center carrying a trans-hexylenic alcohol side chain and a six-membered lactone ring. The putative new PKS-NRPS hybrid gene cluster (*cls*), harboring five genes, was proposed to encode the production of these metabolites, based on ^13^C labeling experiments [109]. A comparison in terms of structure-activity relationships suggests that the presence of a hydroxy group at C-8 may be essential for the bioactivity of cladosin L and other products of the series [146]. The remarkable biosynthetic aptitude of strain SW67 is integrated by a new steroid, namely 3α-hydroxy-pregn-7-ene-6,20-dione, which represents the first compound with a pregnane scaffold isolated from a natural source [145].

Finally, a gorgonian-derived strain (RA7-1) of unidentified species was reported as a prolific producer of 12-membered macrolides and a new bicyclic lactam named cladosporilactam A, which represents the first example of 7-oxabicyclic[6.3.0]lactam obtained from a natural source [102]. Additional novel compounds belonging to the group of macrolides, namely sporiolides A and B, were isolated as products of another unidentified strain (L037) isolated from a brown alga [67].

Besides the few structural revisions mentioned above, some nomenclatural issues have occasionally arisen in the intensive research carried out in the field. For example, the same name was given to different chemical structures (e.g., cladosporiumin I) (Table 3). In the case of cladosin L, two structurally different compounds were given this name in two papers published almost contemporarily, reporting on secondary metabolites of isolates of *C. sphaerospermum* of marine and terrestrial origin [146],[152]; cladosin L considered in the first manuscript is the product of the above-mentioned strain SW67, which is characterized by a 1-amino-3,5-dihydroxyhexylidenyl side chain linked with C-3 to the 2,4-pyrrolidinedione scaffold, instead of a para-disubstituted phenol linked with a double bond at C-3/C-6 [146]. These cases of homonymy were resolved by assigning the suffix ‘*bis*’ to the compounds that were subsequently characterized, as indicated in Table 3. Moreover, we disregarded the report on the ESI-LC-MS analysis of an extract of a strain of *C. cladosporioides* [77], merely providing a list of non-natural compounds or frequently occurring contaminants.

## 5. Secondary metabolites: Bioactivities

The variation in structures and biosynthetic models is reflected by many bioactive properties (Table 4), even if this aspect of the characterization of natural products has not been investigated in all the findings listed in Table 3.

**Table 4.** Bioactive properties of secondary metabolites of *Cladosporium* isolates from marine organisms. AA: anti-adipogenic; B: antibacterial; C: cytotoxic; D: antidiabetic; F: antifungal; I/AS: insecticidal and toxic to *Artemia salina*; LL: lipid lowering; PTP: protein tyrosine phosphatase inhibitor; R: renoprotective against cisplatin-induced kidney cell damage.

Compound	B	F	C	LL	PTP	I/AS	AA	R	D	Ref.
Coniochaetones A, K (1, 3)			√							[116]
*iso*-Cladospolide B (5)	√			√						[102]
11-Hydroxy-*γ*-dodecalactone (6)				√						[117]
(2*S*)-7,4′-Dihydroxy-5-methoxy-8-(*γ,γ*-dimethylallyl)-flavanone (19)					√					[98]
Cladosporamide A (22)					√					[98]
Dendrodolides A, C, M (26-28)	√			√						[102]
Sporiolide A (35)	√	√		√						[67]
Sporiolide B (36)	√			√						[67]
Cladosporol C (38)	√		√							[71]
Cladosporols F, G *bis*, I, J (39, 40, 42, 43)	√									[71]
Cladosporol H (41)	√		√							[71]
Citreoviridin A (61)						√				[83]
Herbarins A, B (62, 63)						√				[83]
Cladospolide E (64)				√						[117]
*seco*-Patulolides A, C (65, 66)				√						[117]
3α-Hydroxy-pregn-7-ene-6,20-dione (67)							√			[145]
Cladodionen (72)			√							[99]
Cladosins B, F, L (73, 75, 76)								√		[146]
Cladosporicin A (77)			√							[109]
Cladosporiumins I *bis*, J *bis* (80, 81)			√							[109]
3,8-Dihydroxy-6-methyl-9-oxo-9*H*-xanthene-1-carboxylate (83)			√							[116]
8-Hydroxy-6-methylxanthone-1-carboxylic acid (86)			√							[116]
8-(Methoxycarbonyl)-1-hydroxy-9-oxo-9*H*-xanthene-3-carboxylic acid (87)			√							[116]
Methyl 8-hydroxy-6-(hydroxymethyl)-9-oxo-9*H*-xanthene-1-carboxylate (88)			√							[116]
Methyl 8-hydroxy-6-methyl-9-oxo-9*H*-xanthene-1-carboxylate (89)			√							[116]
Sumiki's acid (105), acetyl Sumiki's acid (91)	√									[86]
Cladopsols A, B (94, 95)									√	[110]
Cladosporilactam A (98)			√							[102]

Antibiotic effects against *Bacillus subtilis* and *Staphylococcus aureus* were documented for the furan derivative Sumiki's acid (5-hydroxymethyl-2-furan carboxylic acid) and its acetyl derivative [86]. These products were reported as metabolites excreted in human urine after having been first discovered as secondary metabolites of several fungi [153]. Extents of antibacterial activities were also reported for the naphthalenones cladosporols C and F–J against *Escherichia coli*, *Micrococcus luteus*, and *Vibrio harveyi* [71], as well as for *iso*-cladospolide B and dendrodolides A, C, and M against a panel of bacteria, including *E. coli*, *S. aureus*, *Staphylococcus epidermidis*, *Bacillus cereus*, *Tetragenococcus halophilus*, *Pseudomonas putida*, *Nocardia brasiliensis*, and *Vibrio parahaemolyticus* [102].

Moreover, weak inhibitory effects against *M. luteus* (concentration of 16.7 µg mL^−1^) were displayed by sporiolides A and B; the first compound also displayed antifungal activity at the same concentration against *Aspergillus niger* and *Candida albicans*, while *Cryptococcus neoformans* and *Neurospora crassa* were inhibited at a reduced concentration (8.4 µg mL^−1^) [67].

In addition, sporiolides A and B exhibited cytotoxicity against murine lymphoma L1210 cells with IC_50_ of 0.13 and 0.81 µg mL^−1^, respectively [67]. Cytotoxic properties have also been reported for cladosporilactam A against HeLa cells, with IC_50_ of 0.76 µM [102], and cladodionen against MCF-7, HeLa, HCT-116, and HL-60 cell lines, with IC_50_ values of 18.7, 19.1, 17.9, and 9.1 µM, respectively [99]. Three new spirocyclic products, namely cladosporicin A, cladosporiumins I *bis*, and J *bis*, were found to induce weak cytotoxic effects on four breast cancer cell lines (Bt549, HCC70, MDA-MB-231, and MDA-MB-468) [109]. Moreover, coniochaetones A and K, along with the related series of xanthone carboxylate compounds, have been demonstrated to possess significant cytotoxicity against the prostate cancer cell lines 22RV1 and C4-2B at 10 µM, with inhibitory effects between 55.8% and 82.1%, respectively, while displaying nearly no cytotoxicity toward the control RWPE-1 prostate epithelial cells [116]. Finally, cladosporol H has shown significant cytotoxicity against A549, Huh7, and LM3 cell lines with IC_50_ values of 5.0, 1.0, and 4.1 µM, respectively, while cladosporol C showed activity against H446 cells with IC_50_ of 4.0 µM [71].

Besides the antibiotic and cytotoxic effects that are indicative of a general bioactivity of the above compounds, more specific properties have been evaluated in several studies, which point out the opportunity for more refined assessments on the profiles and the mechanisms of action of *Cladosporium* secondary metabolites.

Insecticidal effects were observed for the known pyrone derivative citreoviridin A, which was responsible for 33% growth inhibition of larvae of the lepidopteran *Spodoptera littoralis* at a concentration of 250 ppm, while herbarins A and B were found to be toxic to *Artemia salina*, inducing consistent mortality rates at doses of 100 µg and 50 µg, respectively [83].

Renoprotective effects derived from the reduction of cisplatin nephrotoxicity were determined for cladosins B and F on LLC-PK1 porcine kidney cells. The effect on cell viability by cladosin B at 100 µM in the presence of cisplatin (25 µM) was similar to the control set with a higher concentration of N-acetylcysteine (500 µM). In this assay, cladosin L had a much lower protective effect, while cladosin C was inactive [146].

Potent lipid-lowering effects in HepG2 liver cancer cells were shown by the hexaketides cladospolide E, *seco*-patulolides A and C, and 11-hydroxy-γ-dodecalactone; particularly, the IC_50_ value of oleic acid-elicited lipid accumulation of the latter compound was lower than the one determined for the blockbuster anticholesterolemic drug lovastatin, used as a control product [117]. Another kind of lipid-lowering aptitude characterizes the steroid 3α-hydroxy-pregn-7-ene-6,20-dione, which was found to substantially inhibit lipid accumulation during adipocyte maturation. In fact, a marker gene (adipsin) was downregulated when the adipocytes were incubated in the presence of this compound; furthermore, the expression of the lipolytic gene adipose triglyceride lipase (ATGL) was promoted, whereas the expressions of the lipogenic genes encoding fatty acid synthase (FASN) and the sterol regulatory element-binding protein 1 (SREBP1) were significantly reduced [145].

Adipose cells are the major targets for peroxisome proliferator-activated gamma (PPAR-γ) agonistic drugs, which have a potential as antidiabetic therapeutics improving lipid metabolism, glucose uptake, and insulin sensitivity. Based on a luciferase reporter assay and docking analysis, the acylic diterpene cladopsol B was prospected as a potential antidiabetic lead functioning as a PPAR-γ partial agonist, which may avoid the side effects of full agonists. Moreover, cladopsol B was found to stimulate glucose uptake in HepG2 cells; in this respect, its efficacy is comparable to rosiglitazone, with minor undesired consequences on lipid accumulation. Taken together, these properties characterize cladopsol B as a molecular model when searching for advanced antidiabetic drugs [110]. Finally, the new tricyclic compound cladosporamide A, along with the known (2*S*)-7,4′-dihydroxy-5-methoxy-8-(γ,γ-dimethylallyl)-flavanone, exhibit modest inhibitory properties toward protein tyrosine phosphatase (PTP); this enzyme is a key negative regulator in the insulin signaling pathway, thereby representing a molecular target for the treatment of type-2 diabetes [98].

## 6. Conclusions

In this review, we highlight the widespread occurrence of fungi of the genus *Cladosporium* as symbiotic associates of marine organisms, as attested by dozens of isolations from species belonging to many phyla of plants and animals in every kind of geographic and climatic context. Pending a more diffuse application of methods for a correct taxonomic identification, many strains in this collection have not been properly classified; hence, it is not possible to point out if any species has a systematic association with certain organisms, playing a defined functional role. Studies on the biosynthetic abilities of these fungi have been more circumstantial, disclosing the production of as many as 106 secondary metabolites belonging to several classes. Among them, the tetramic acids and the naphthalenones seem to be particularly represented, including several products typical of the genus *Cladosporium*. Overall, about 27% of the compounds are novel, pointing out the relevance of these fungi as a source of chemodiversity deserving more circumstantial assessments on the bioactivities and the perspectives for biotechnological exploitation.

## Use of AI tools declaration

The authors declare they have not used Artificial Intelligence (AI) tools in the creation of this article.
